# Reducing crude protein content in the diet of lactating dairy cows improved nitrogen-use-efficiency and reduced N excretion in urine, whilst having no obvious effects on the rumen microbiome

**DOI:** 10.1186/s40104-025-01240-7

**Published:** 2025-08-09

**Authors:** Agalu W. Zeleke, Nicholas J. Dimonaco, Katie Lawther, Anna Lavery, Conrad Ferris, Jon Moorby, Sharon A. Huws

**Affiliations:** 1 School of Biological Science and Institute for Global Food Security (IGFS), at Queen’s University Belfast, Northern Ireland, UK; 2 Agri-Food and Biosciences Institute, Livestock Production Sciences Branch, Large Park, Hillsborough, Northern Ireland, County Down UK; 3 Institute of Biological, Environmental and Rural Sciences at Aberystwyth University, Wales, UK

**Keywords:** Dairy cows, Dietary crude protein, Nitrogen excretion, Nitrogen-use-efficiency, Rumen microbiome

## Abstract

**Background:**

Nitrogen-Use-Efficiency (NUE) in lactating dairy cows, defined as milk nitrogen (N) output as a proportion of N consumed, is low, with the majority of excess N excreted in manure. Excreted N can be lost to the environment as ammonia gas leading to environmental acidification and nutrient enrichment of sensitive habitats, and to watercourses contributing to aquatic eutrophication. While there is much evidence that NUE can be improved by reducing the crude protein (CP) content of dairy cow diets, the long-term impacts of feeding lower protein diets on cow performance and the rumen microbiome are less well understood. This study examined the effects of reducing the CP contents of dairy cow diets on cow performance, NUE, the relationship between NUE and residual feed intake (RFI), and the rumen microbiome.

**Results:**

Dietary CP content did not affect feed intake, milk yield or milk composition (*P* > 0.05), except for milk urea N (MUN), which increased with increasing diet CP content (*P* < 0.05). The mean NUE was 34%, 34% and 31% for the LCP (low-protein, 15%), MCP (medium-protein, 16%), and HCP (high-protein, 17%) diets, respectively. RFI was negatively correlated with NUE (*r* = −0.57, *P* < 0.001). The rumen ammonia-N concentrations increased with increasing dietary CP; however, the ruminal pH and volatile fatty acid (VFA) content of the rumen fluid remained constant. Predicted urinary N excretion was greater in the HCP and MCP diets than in the LCP diet. Reducing dietary CP content in dairy cow diets did not affect microbial composition, diversity and functional profiles. The family Bacteroidaceae was more abundant in HE (high-efficiency) cows, whereas the Methanobacteriaceae and the genus *Methanobrevibacter* were more abundant in LE (low-efficiency) cows. Additionally, propanoate metabolism, cysteine and methionine metabolism and amino acid biosynthesis pathways were more abundant in HE cows, whilst the methane (CH_4_) metabolism pathway was upregulated in LE cows.

**Conclusions:**

The results demonstrate that diet CP can be reduced with no loss in cow performance, but with an associated reduction in N excretion. The abundance of microbial populations differed between low and high efficiency cows, which may contribute to the differences in efficiency observed.

**Graphical abstract:**

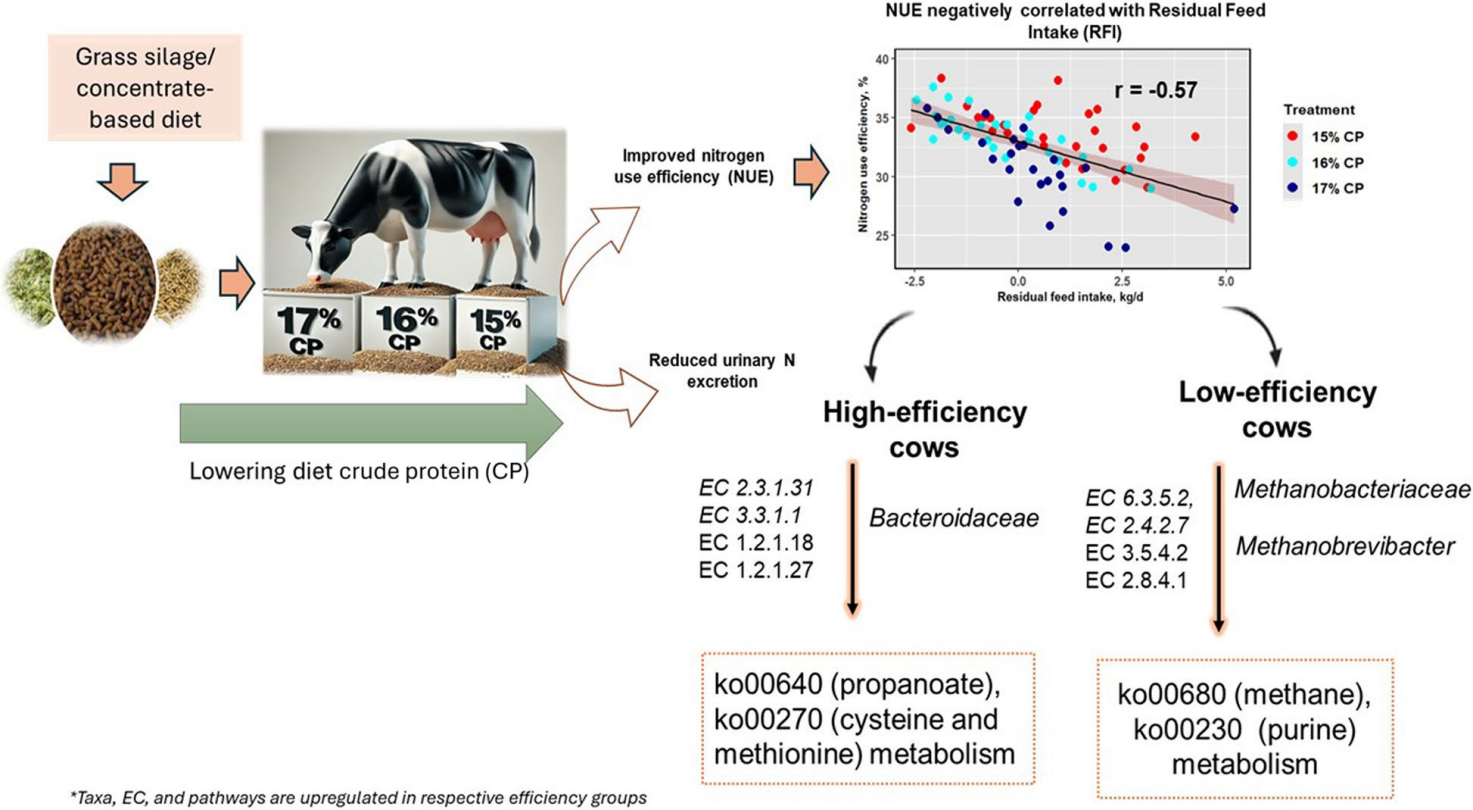

**Supplementary Information:**

The online version contains supplementary material available at 10.1186/s40104-025-01240-7.

## Introduction

Nitrogen-Use-Efficiency (NUE) in lactating dairy cows, defined as milk nitrogen (N) output as a proportion of N consumed, is low, with the majority of consumed N excreted in urine and faeces [1, 2]. For example, in a review of studies involving dairy cows offered primarily grass silage-based diets, Yan et al. [3] observed that 22% of N consumed was excreted in milk, whereas 43% and 29% were excreted in urine and faeces, respectively. Actual NUE on commercial dairy farms can be extremely variable, ranging from 16% to 36% [4], with Craig et al. [5] calculating a mean NUE of 30% in a recent study involving 26 Northern Ireland dairy farms. Nitrogen losses in manure create a number of challenges. For instance, N losses via volatilisation, primarily as ammonia gas, can lead to acidification of water and soils [6, 7], terrestrial eutrophication and loss of biodiversity within sensitive habitats [8]. Nitrogen, primarily as nitrates, can also be lost to watercourses via leaching, contributing to aquatic eutrophication [9]. Additionally, N (protein ingredients) normally represents the highest cost component of dairy cow diets [10].

While improving NUE is important for improving the long-term sustainability of milk production systems, dairy cows have traditionally been overfed crude protein (CP) in an attempt to improve milk production [11]. For example, a meta-analysis by Ipharraguerre and Clark [12] observed a daily milk yield response of 0.94 kg when the dietary CP content increased from 15% to 16% (DM basis), decreasing to 0.42 kg when diet CP content increased from 19% to 20%. However, given that higher CP levels are generally associated with reduced NUE, there is now considerable interest in reducing the CP content of dairy cow diets. A number of studies have examined the impact of reducing the CP content of dairy cow diets. For example, when the CP content of the diet in early lactation was reduced from 17.4% to 15.4%, dry matter intake (DMI) was unaffected while milk yield was reduced [13]. A similar effect was reported by Law et al*.* [14] when dietary CP was reduced from 17.3% to 14.4% during the first 150 d of lactation. In contrast, Reynolds et al*.* [15] found a reduction in DMI when diet CP was reduced from 18% to 16%, while milk yield was unaffected.

Contrasting results in these studies may reflect the fact that cows do not have a requirement for ‘crude protein’ per se, but rather have a requirement for amino acids, with the latter supplied either from protein that bypasses the digestive process in the rumen or from microbial protein synthesised in the rumen. While the impact of diet on the rumen microbiome has been extensively studied, the impact of the rumen microbiome on performance is less so but has gained attention more recently. For instance, Jami et al. [16] found a positive association between milk-fat yield and the ratio of Firmicutes*/*Bacteroidetes, while Wu et al. [17] found that *Prevotella* was more abundant in the rumen of cows with high milk fat and protein content compared to those with low milk fat and protein content. Similarly, Bainbridge et al*.* [18] reported a moderate association between rumen bacterial communities and milk production, milk protein, and milk fat synthesis.

While these studies suggest that rumen microbiota may impact cow performance, our understanding of these relationships is limited. Specifically, the relationships between the rumen microbiota and metrics of efficiency, such as NUE and residual feed intake (RFI: the difference between actual feed intake and predicted feed intake), are poorly understood [19]. However, in a recent study, Li et al. [20] reported differences in the rumen microbial community between cows differing in NUE, with communities in cows with low NUE being more diverse than those in medium and high NUE cows. Similarly, with regards to RFI, it has been reported that cows with lower RFI values utilise protein more efficiently than those with higher RFI values [21], although published evidence is not widespread.

Developing an improved understanding of the relationships between NUE, RFI, and the rumen microbiome will help optimise the efficiency and sustainability of dairy production systems while minimising environmental impacts. Consequently, this study aimed to provide novel data on the effects of diet CP content on the relationships between NUE, RFI, and the rumen microbiome.

## Materials and methods

### Animals and diets

This study was conducted at the Agri-Food and Biosciences Institute (AFBI), Hillsborough, Northern Ireland. All experimental procedures were carried out under an experimental licence given by the Northern Ireland Department of Health, Social Services, and Public Safety in compliance with the Animals (Scientific Procedures) Act 1986. Holstein dairy cows (24 primiparous and 66 multiparous (mean calving date, 15 November 2020, ± 8.4 d)) were allocated to one of three treatments prior to calving on the basis of the expected calving date, parity (primiparous and multiparous cows allocated separately), genetic potential for milk yield, fat yield, protein yield (kg), fat% and protein%, previous lactation milk yield (305-d), milk fat content, and milk protein content (multiparous cows only), and body weight (BW) measured during the 2-week period pre-calving. Following calving, cows were transferred to an experimental free-stall house where they had access to individual cubicles beds (free-stalls) that were fitted with rubber mats and bedded with sawdust. Cows had access to fresh water at all times. During the first 7 d following calving, all cows were offered a common diet containing 160 g CP/kg DM to allow collection of ‘baseline’ rumen fluid samples. From d 8 to 180 post calving, cows were offered one of three dietary treatments which were designed to contain either 15%, 16% or 17% CP/kg DM. All three diets were formulated to supply ≥ 100% of the cows estimated metabolisable protein (MP) requirements based on equations within Feed-into-Milk (FiM), the current feed rationing system for dairy cows within the United Kingdom (UK).

Rations were offered as partial mixed rations comprising grass silage, concentrates (mixed in equal proportions (50:50) on a DM basis) and chopped straw, with the latter included in the ration to achieve a target intake of 0.32 kg/cow/d. The concentrates offered with each treatment differed in ingredient and chemical composition, so as to achieve the different total diet CP levels required. Variation in silage composition during the course of the study required the ingredient composition of the concentrates offered to be adjusted on a number of occasions so as to maintain total diet CP levels. In addition, throughout the experiment all cows were offered a common ‘commercial concentrate pellet’ during milking to aid cow flow onto the milking parlour, 0.25 kg/cow at each milking. The ingredient composition of this parlour concentrate was as follows (g/100 g fresh basis): wheat, 17.4; maize meal, 17.5; maize distillers, 8.5; maize gluten, 11.0; sugar beet pulp, 6.1; soyabean meal (high protein), 8.6; soya hulls, 17.45; sugarcane molasses (liquid), 8.0; palm fatty acid distillate, 0.95; mineral/vitamin premix, 4.5. The mean ingredient composition of each of the three experimental concentrates offered, weighted for the mean number of feeding days that each concentrate formulation was offered for, is presented in Table S1. The ingredient compositions of the individual diet components are presented in Table S2.

Rations were prepared daily at ~0900 to 1000 h using a paddle-type diet feeder wagon (Redrock, Armagh, NI, UK) and offered ad libitum at 107% of the previous day’s intake, while uneaten ration was removed the following day at approximately 0800 h. Following mixing rations were transferred from the diet feeder to a series of feed boxes mounted on weight scales (Controlling and Recording Feed Intake, Bio-control, Rakkestad, Norway), with cows accessing food in these boxes via an electronic identification system, enabling individual cow intakes to be recorded daily. Dry matter intakes were determined based on the DM content of individual ration components. A full description of the methodology used to record feed intakes, milk production, milk composition and bodyweight has been provided by Lavery et al. [22]. The full details of cow management, cow measurements, diet preparation, sampling of feeds and lab analysis of feeds have been presented by Lavery et al. [22].

### Rumen fluid sampling

On d 7 (± 1 d) following calving (when all cows were being offered a common diet: baseline rumen sample), and at weeks 4, 8, 12, and 20 post-calving (± 3 d) rumen fluid samples were collected via an oro-ruminal probe (Ruminator; Profs-products, Wittibreut, Germany). The oro-ruminal probe was inserted into the oesophagus, and the cow was allowed to swallow the probe until the pre-marked area on the tubing was reached. A hand pump connected to a collection jar was attached, and an initial rumen fluid sample of approximately 100 mL was obtained and discarded. The handpump/collection jar was disconnected and washed under cold running water and then re-connected and a second sample (approximately 300 mL) was collected. The handpump/collection jar was detached, and the oro-ruminal probe was slowly removed from the cow. The rumen fluid was decanted into a 50-mL Falcon tube and a 2-mL microcentrifuge tube, flash frozen in liquid nitrogen, and stored at −80 °C until analysis.

### Rumen fermentation

Rumen fluid samples were defrosted at room temperature for 40 min, and the pH of the rumen fluid was determined via a calibrated pH meter (Mettler Toledo, FiveGo Portable F2 pH/mV Metre, USA). A 30 mL sample of rumen fluid was centrifuged at 20,000 × *g* at 4 °C for 10 min and 2 mL of the supernatant in a 2-mL microcentrifuge tube was stored at −80 °C for subsequent VFA analysis. A total of 25 µL of the supernatant was used to determine NH_3_-N concentration using the phenol‒sodium hypochlorite colorimetric technique [23], and the light absorbance value at 630 nm was measured using a BMG Labtech CLARIOstar plate reader (version 3.32 UK). The ammonia standard solution was prepared using Chaney and Marbach [24] technique, which involves dissolving 63 mg of ammonium chloride in 100 mL of distilled water and measuring the standard curve using 0, 5, 10, 15, 20, and 25 µL of the ammonia standard concentration diluted with the appropriate amount of distilled water and 200 µL of each solution was utilised for colorimetric ammonia measurement.

For VFA analysis, a 2 mL rumen fluid sample was defrosted and centrifuged at 20,000 × *g* at 4 °C for 10 min and 200 µL of the supernatant from each sample tube was transferred into a new 2-mL microcentrifuge tube labelled with the corresponding sample ID. This subsample was diluted with 800 µL of deionised water while 10 µL of stability preservative (saturated mercuric chloride) was spiked into each sample and the microcentrifuge tubes were then stored at 4 °C until reagents were prepared for the next step. Finally, 200 µL of diluted sample solution, 200 µL of internal standard and 600 µL of deionised water (final volume of 1 mL) were transferred into 2-mL gas chromatograph vial tubes and vortexed for 5 s and the VFA concentration was determined using a Scion 456 gas chromatograph for on-column injection and flame ionisation detection in accordance with the manufacturer's instructions.

### Mathematical calculations

Nitrogen-use-efficiency (%) was calculated as: $\lbrack(\mathrm{total}\;\mathrm{milk}\;\mathrm N\;(\mathrm g/\mathrm d)/\mathrm N\;\mathrm{intake}\;(\mathrm g/\mathrm d)\rbrack\;\times\;100$ [25].

Nitrogen excretion in urine was predicted according to Kohn et al*.* [26].

$$\mathrm{Predicted}\;\mathrm{urine}\;\mathrm N\;\mathrm{output}\;\left(\mathrm g/\mathrm d\right)=15.1\times\mathrm{milk}\;\mathrm{urea}\;\mathrm N\;\left(\mathrm{mg}/\mathrm{dL}\right)+27.8$$

Faecal N output was estimated according to Bahrami-yekdangi et al*.* [27] using the following equation:

$$\mathrm{Predicted}\;\mathrm{faecal}\;\mathrm N\;\mathrm{output}\;\left(\mathrm g/\mathrm d\right)=\mathrm N\;\mathrm{intake}\;\left(\mathrm g/\mathrm d\right)-\;\mathrm{predicted}\;\mathrm{urine}\;\mathrm N\;\left(\mathrm g/\mathrm d\right)-\mathrm{milk}\;\mathrm N\;\left(\mathrm g/\mathrm d\right)$$

Residual feed intake was estimated as:

$$\mathrm{RFI}\;\left(\mathrm{kg}/\mathrm d\right)=\mathrm{actual}\;\mathrm{DMI}\;\left(\mathrm{kg}/\mathrm d\right)-\mathrm{predicted}\;\mathrm{DMI}\;\left(\mathrm{kg}/\mathrm d\right)$$

Within each treatment, the RFI was estimated for each cow during four 5-week periods (weeks 2–6, 6–10, 10–14 and 18–22 post-calving), with these periods centered on the rumen sampling occasions at weeks 4, 8, 12 and 20 post calving, as follows. Across each of these 5-week periods, the mean daily total metabolisable energy requirements were calculated for each cow (sum of requirements for milk, maintenance, activity, and body weight gain/loss) using the energy equations within the FiM. The total ME requirements were then converted to the expected dry matter intake by dividing by the ME density of the diet offered (derived as actual silage DMI × silage ME content (predicted using NIRS) plus actual concentrate DMI × concentrate ME density (estimated from the ME density of individual concentrate components, as contained within the FIM database), plus actual straw DMI × ME density (assumed as 6.5 MJ/kg DM, as per FIM database)) by the total DMI. The residual feed intake during each of the four 5-week periods was then estimated for each individual cow as the difference between actual DMI (kg/d, mean for each 5-week period) and predicted DMI (kg/d, mean for each 5-week period). The mean RFI was then determined for each cow over the four 5-week periods, and primiparous and multiparous cows then ranked from highest to lowest RFI within each treatment. The two most and least efficient primiparous cows (four in total) were then selected from each treatment. Similarly, four of the most and least efficient multiparous cows (eight in total) were then selected from each treatment (with cows selected so that each sub-group was largely balanced for parity). In total, 36 dairy cows (12 cows from each treatment, 6 HE and 6 LE cows) were selected, representing a total of 180 rumen samples (3 treatments, 5 sampling periods, 12 cows per treatment). Of the 180 rumen samples, three samples were lost during processing, with 177 samples included in the final microbiome analysis using the shotgun metagenomic sequencing approach.

### Microbiome analysis

DNA extraction was carried out using the Qiagen DNeasy PowerSoil Pro Kit following the manufacturer’s protocol. Firstly, 500 µL of defrosted rumen fluid was centrifuged at 15,000 × *g* for 5 min. Then, the supernatant was discarded, and the pellet was re-suspended in 800 µL of CD1 solution, briefly vortexed, and poured into a PowerBead Pro tube. Homogenisation and mechanical cell disruption were achieved by bead-beating for 1 min at 5.5 m/s × 3 times in FastPrep (MP Biomedicals, Solon, OH, USA), with incubation on ice between each cycle. The full details of the protocol can be found at Qiagen protocol. A total of 75 µL of ZymoBIOMICS™ Microbial Community Standard (D6300) was included as a positive control. During each extraction, a kit reagent was used as a negative control to monitor contamination. DNA quantification was performed using Nanodrop and a Qubit dsDNA HR assay kit (Invitrogen) on a DeNovix QFX fluorometer. All samples were adjusted to ensure that the final DNA input for library preparation was between 100 and 500 ng. For samples with DNA concentrations above 250 ng/µL, the DNA was diluted with nuclease-free water to a concentration of 50–250 ng/µL, so that 2 µL of DNA input fell within the required range. For samples with DNA concentrations below 50 ng/µL, the input DNA volume was increased to 3 µL to achieve the required total DNA amount (100–500 ng). Then, shotgun metagenomic libraries were prepared using 2–3 µL of genomic DNA with a total input of 100–500 ng of DNA using the Illumina DNA Prep Kit (Illumina, San Diego, CA, USA). The library prep kit reagent was included as a negative control, and the DNA extracted from the ZymoBIOMICS™ microbial community standard (as described above) was used as a positive control. Finally, all individual 150-bp paired-end libraries were transferred to the Genomics Core Technology Unit (GCTU, Queen’s University Belfast, Northern Ireland), where library quality and concentration were assessed on an Agilent 2100 Bioanalyzer and metagenomic sequencing was performed using the Illumina Nova Seq 6000 S4 300 flow cell platform (Illumina Inc., San Diego, CA, USA, 150 bp paired-end sequencing).

A total of 177 rumen fluid DNA samples from 36 dairy cows (selected based on RFI, as described above) were sequenced, producing a total of 9,574,699,324 raw reads, with an average of 53,192,774 reads per sample. All tools were used with default parameters unless otherwise specified. A Fast QC report was generated to identify overall sequence quality and distribution. Then, Trimmomatic (v0.39) with the following parameters: *ILLUMINACLIP: TruSeq3-PE-2:2:30:10 LEADING:3 TRAILING:3 SLIDINGWINDOW:4:20 MINLEN:50* was used to trim low-quality bases and adapters and identify reads with corresponding pairs (dropping reads if one pair was missing during sequencing). The reads were trimmed at any site of four sequential bases with a ‘*SLIDINGWINDOW’* if the quality dropped below Q20. Any reads reporting a quality score of < Q20 and short reads less than 50 bp (after trimming) were discarded.

The ‘cleaned’ reads from each of the samples were then mapped to the human (*GCF_000001405.40_GRCh38.p14*), cow (*GCF_002263795.2_ARS-UCD1.3*) and sheep (*GCF_016772045.1_ARS-UI_Ramb_v2.0*) reference genomes using Bowtie2 (v2.5.1) [28] with default parameters to identify the level of contamination and remove human contaminants, and host-associated DNA. Reads that aligned concordantly with references were considered contaminants and discarded. We obtained a total of 9,032,179,290 clean reads, with an average of 50,178,774 reads from each sample. These quality-checked, and decontaminated reads were metagenomically assembled using metaSPAdes (spades v3.15.5) [29], producing 3,128,357 contigs. The resulting contigs that were greater than or equal to 2,500 bp in length underwent taxonomic classification using Kraken2 (v2.1.3) [30] using the ‘PlusPFP’ (Standard plus RefSeq protozoa, fungi and plant) precomputed database (https://genome-idx.s3.amazonaws.com/kraken/k2_pluspfp_20230605.tar.gz) with default parameters. Additionally, Pyrodigal (v3.0.1) [31, 32] was used to predict protein coding genes from the same contigs which were subsequently annotated with eggNOG-mapper (v2.1.12) [33] using the eggNOG 5.0 database [34]. Finally, Bowtie2 was applied to map the cleaned reads back to the assembled contigs to count the percentage of reads assigned to each taxa that had been identified by Kraken2. The bedtools intersect (v2.31.1) [35] was then used to report reads that were specifically mapped to regions of the contigs previously identified by Pyrodigal as containing protein coding genes. The outputs from these tools were then combined into Tab-separated files with the full taxonomic and functional classification and read mapping numbers for each sample by the MetaPont package.

### Statistical analysis

Data analysis was performed in R (version 4.4.0) using RStudio (version 2024.4.1.748). Data for milk yield, milk composition, feed intake, NUE, N excretion and rumen fermentation (weekly measurements), with rumen fluids collected at weeks 4, 8, 12, and 20, were analysed using a mixed-effects model (autoregressive model of order 1) fitted by Restricted maximum likelihood (REML) estimation method, with weekly measurements taken from the same animal included as a repeated measure (weeks 1–26) and cow as the random effect, treatment (LCP, MCP, HCP) as a fixed effect and lactation number (1, 2, 3, 4) fitted as an additional fixed effect (covariate for all variables) using the following formula:

$$y_{tjk}=\mu+T_t+W_j+{\left(T\times W\right)}_{tj}+C_k+\varepsilon_{tjk}$$

where *y*_*tjk*_ = the response variable, *μ =* the overall mean, *T*_*t*_ = the main effect of treatment *t* (*t* = 1, 2, 3), *W*_*j*_ = the main effect of sampling week *j* (*j* = 1–26), (*T* ×*W*)_*tj*_ = the interaction effect of treatment × sampling week, *C*_*k*_ = the random effect of cow *k*, and *ε*_*tjk*_ = the residual error term. 

The autoregressive model of order 1 was fitted to model correlations between weeks. If any of the week, treatment or interaction between the two variables was significant (*P* < 0.05), then Tukey’s Honest Significant Difference (HSD) test was used for post-hoc pairwise comparisons of the levels of individual fixed effects. The ‘lmodel2*’* function in R was used to fit a type II linear regression (Reduced Major Axis [RMA] regression) to estimate the relationship between NUE and RFI using the following formula:

$$y_i=ax_i+b+\varepsilon_i$$

where *y*_*i*_ = the response variable, *x*_*i*_ = the observed value of the independent variable for the *i*-th observation, *a* = the slope of the regression line, *b* = intercept of the regression line, ε_*i*_ = the residual error term.

The ‘cor.test’ function in R was used to calculate correlation coefficients between NUE and RFI and to test their significant differences. The microbiome count data was normalised using the trimmed mean of M-values (TMM) method, as described in earlier studies [36, 37]. This technique employs the ‘calcNormFactors’ function from the edgeR package (version 4.2.0) in R to adjust for differences in library size and composition biases inherent in count data. Microbial community diversity was assessed using alpha diversity indices, such as richness (Chao1), evenness (Pielou's), and diversity (Inverse Simpson), which were computed using the vegan package (version 2.6.4) in R [38]. The Kruskal‒Wallis test was employed to analyse differences across diets and periods, while the Wilcoxon rank‒sum test was used to compare efficiency groups. In addition, beta-diversity was performed with principal coordinate analysis (PCoA) based on Bray–Curtis dissimilarity matrices at phylum, family and genus level using the vegan package in R and the significant effects of each variable were assessed using permutational multivariate analysis of variance (PERMANOVA), which was conducted in R using the ‘adonis2’ function in the Vegan package, with 999 permutations and Benjamini‒Hochberg (BH) correction was employed to adjust *P-*values for multiple testing. To enhance statistical robustness and reduce analysis noise, a threshold of 100 total sums of read counts across all samples per dataset was applied to remove low abundance reads for downstream analysis. Linear discriminant analysis (LDA) with linear discriminant effect size (LefSe) was employed for differential abundance analysis of both the taxonomic and functional parameters [39–41], using an LDA score > 2 and *P* < 0.05 were used as cut-offs. Differences at 0.05 < *P* < 0.10 were considered as a trend toward significance.

## Results

### Feed intake and lactation performance

There were no differences among treatment groups for DMI (*P* > 0.05, Table 1). Similarly, dietary CP content did not affect milk yield, milk fat, protein, lactose and energy content, or energy corrected milk (*P* > 0.05). However, MUN concentration increased with increasing dietary CP content (*P* < 0.001). Cows offered the HCP and MCP diets had MUN concentrations that were 27.6% and 15.3% higher than those offered the LCP diet, respectively. Bodyweight did not vary in response to diet CP concentration (*P* > 0.05). All production parameters varied over time (*P* ≤ 0.001), but there was no treatment × week interactions for any of the tested variables, except for milk protein content (*P* < 0.001, Table 1).

**Table 1:** Effects of diet crude protein content and sampling period on milk production, milk composition, feed intake, nitrogen-use-efficiency and nitrogen excretion by lactating dairy cows

Parameters	Treatment (T)^1^			SEM^3^	*P*-value^2^		
	LCP	MCP	HCP		T	Week (W)	T × W
Dry matter intake, kg/d	23.2	23.3	23.8	0.29	0.082	< 0.001	0.862
Milk yield, kg/d	35.7	37.1	36.3	0.97	0.536	< 0.001	0.995
Fat, g/kg	44.9	44.6	44.7	0.47	0.746	< 0.001	0.593
Protein, g/kg	34.4	34.8	34.9	0.28	0.240	< 0.001	< 0.001
Lactose, g/kg	48.6	48.5	48.5	0.13	0.812	< 0.001	0.602
Milk energy, MJ/kg	3.33	3.33	3.37	0.02	0.642	< 0.001	0.846
ECM, kg/d	38.8	40.4	39.3	0.72	0.631	< 0.001	0.542
MUN, mg/dL	9.7^a^	11.5^b^	13.4^c^	0.18	< 0.001	< 0.001	0.094
BW, kg	632	636	651	10.2	0.201	< 0.001	0.929
Total N intake, g/d	568^a^	602^b^	648^c^	7.18	< 0.001	< 0.001	0.623
N output in milk, g/d	194	203	202	4.67	0.110	0.001	0.556
NUE, %	34.3^b^	34.1^b^	31.2^a^	0.004	< 0.001	< 0.001	0.659
Urinary N^4^, g/d	174.9^a^	201.5^b^	231.0^c^	3.20	< 0.001	< 0.001	0.419
Urinary N/N intake, %	31.3^a^	34.0^ab^	36.3^b^	0.61	0.003	< 0.001	0.283
Faecal N^5^, g/d	201.2	199.9	212.9	3.22	0.597	< 0.001	0.718
Faecal N/N intake, %	34.9	32.6	33.0	0.55	0.157	< 0.001	0.621
Total N excretion, g/d	376.1^a^	401.5^a^	443.0^b^	3.71	< 0.001	< 0.001	0.430
N excretion/N intake, %	66.3^a^	66.6^a^	69.3^b^	0.32	< 0.001	< 0.001	0.352

*ECM* Energy corrected milk, *MUN* Milk urea nitrogen, *BW* Bodyweight, *NUE* Nitrogen use efficiency

^a–c^ Differences in superscript indicate significance at *P* < 0.05

^1^Treatments: LCP (15% CP), MCP (16% CP), HCP (17% CP)

^2^*P*-values: T = main effect of treatment; W = main effect of week; T × W = interaction effect of treatment and week

^3^*SEM* Standard error of the mean

^4^Urinary N output predicted using equations from Kohn et al*.* [26]

^5^Faecal N output predicted using equations from Bahrami-yekdangi et al*.* [27]

### Nitrogen-use-efficiency, N excretion, RFI and rumen fermentation

**Fig. 1:**
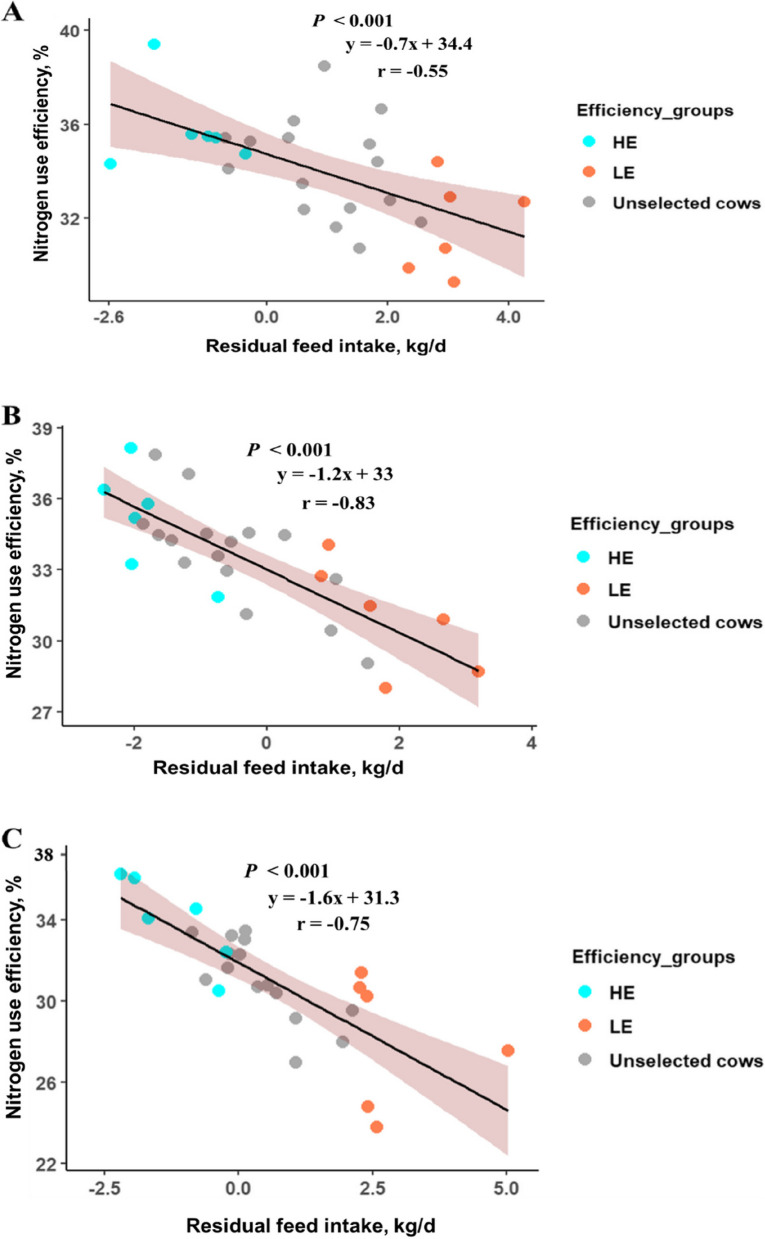
The relationships between NUE and RFI for cows offered diets containing (**A**) LCP (15% CP), (**B**) MCP (16% CP) and (**C**) HCP (17%). Cows within the high (HE) and low (LE) efficiency groups were selected on the basis of RFI for microbiome analysis (*n* = 177). Unselected cows: cows which were not considered for microbiome analysis

Across all individual cows in the experiment, NUE ranged from 24% to 39%, while the mean N usage efficiencies were 34%, 34%, and 31% for the LCP, MCP, and HCP protein diets, respectively (*P* < 0.001, Table 1). Cows on the HCP diet had a lower NUE than those on the LCP and MCP. The predicted urinary N output increased with increasing dietary CP content (*P* < 0.001), while predicted N excreted in faeces was unaffected (*P* > 0.05). When expressed as a percentage of N intake, urinary N excretion with LCP was lower than that with HCP (*P* < 0.001), while UN excretion did not differ between LCP and MCP (*P* > 0.05). While NUE, and N excreted in urine and faeces varied over time (*P* < 0.001), no treatment × week interactions were observed (*P* > 0.05). The mean residual feed intake differed between the three treatment groups (0.9, −0.3 and 0.4 kg/d for the LCP, MCP and HCP diets, respectively: *P* = 0.017), while the RFI was negatively correlated with NUE (*r* values of −0.55, −0.83 and −0.75 for the LCP, MCP and HCP diets, respectively: *P* < 0.01, Fig. 1). An additional file contains all raw data used for this analysis (see Additional file 1: Production and fermentation data).

Dietary CP levels had no effect on the molar concentrations of any of the VFAs measured in the rumen fluid or on total VFA concentrations (*P* > 0.05, Table 2). However, the concentrations of all VFAs varied over time. For example, at sampling periods 4, 8, 12 and 20 acetate concentrations were 1.79, 2.70, 1.01 and 3.16 mmol/L, propionate concentrations were 0.73, 1.04, 1.01and 1.13 mmol/L and butyrate concentrations were 0.59, 0.82, 0.77, and 0.91 mmol/L, respectively. There was no treatment × time interaction for any of the VFA measured (*P* > 0.05). The mean ammonia-N concentrations in the rumen fluid were 19.9, 20.5, and 31.1 mg/L of rumen fluid for cows offered LCP, MCP, and HCP, respectively (*P* < 0.01), with concentrations for HCP being higher than for LCP and MCP (*P* < 0.05). Rumen pH was not affected by diet (*P* > 0.05), although pH changed over time (7.1, 6.8, 6.9 and 6.7 in sampling periods 4, 8, 12, and 20, respectively). There was no treatment × time interaction for pH (*P* > 0.05).

**Table 2:** Effects of diet CP concentrations and sampling period on the rumen fermentation parameters of lactating dairy cows

Parameters	Treatment (T)^1^			SEM^3^	*P*-value^2^		
	LCP	MCP	HCP		T	Week (W)	T × W
Total VFA, mmol/L	98.0	100.0	101.4	1.30	0.567	< 0.001	0.851
Acetate, mmol/L	55.2	57.0	57.6	0.73	0.375	< 0.001	0.884
Propionate, mmol/L	21.2	21.2	21.7	0.37	0.825	0.002	0.756
Butyrate, mmol/L	16.8	17.0	17.2	0.29	0.856	0.003	0.900
Isobutyrate, mmol/L	1.0	1.0	1.1	0.01	0.953	< 0.001	0.946
Valerate, mmol/L	2.1	2.1	2.2	0.05	0.875	0.012	0.638
Isovalerate, mmol/L	1.7	1.7	1.7	0.04	0.890	0.005	0.568
A:P^4^	2.7	2.7	2.7	0.04	0.740	< 0.001	0.914
Ammonia-N, mg/L	19.9^a^	20.5^a^	31.1^b^	1.24	< 0.001	0.001	0.158
Ruminal pH	6.9	6.9	6.9	0.01	0.638	< 0.001	0.851

^a,b^ Differences in superscript indicate significance at *P* < 0.05

^1^Treatment: LCP (15% CP), MCP (16% CP), HCP (17% CP)

^2^ *P*-values: T = main effect of treatment; W = main effect of week; T × W = interaction effect of treatment and week

^3^*SEM* Standard error of mean

^4^*A:P* Acetate to propionate proportion

### Taxonomy and diversity of rumen microbiota

Microbial community composition in positive controls in our data aligns with the expected values from the ZymoBIOMICS™ microbial community standard and is presented in Fig. S1. The rumen metagenome taxonomic data in this study consisted of 90.86% bacteria, 6.95% eukaryotes, 1.98% archaea, and 0.21% viruses. Within the bacterial domain, there were 46 phylum, 99 classes, 211 orders, 506 families and 1,890 genera. The archaeal domain comprises of nine phylum, 17 classes, 30 orders, 45 families and 141 genera. Within the Eukaryota domain, there were 15 phylum, 26 classes, 58 orders, 89 families and 153 genera. The virus domain comprised 13 phylum, 22 classes, 28 orders, 69 families and 229 genera. The domain archaea were dominated by the phylum Euryarchaeota (99.31%), the family Methanobacteriaceae (93.11%), and the genus *Methanobrevibacter* (89.93%). The dominant bacterial phylum included Bacteroidota (49.33%) and Bacillota (24.04%), followed by Pseudomonadota (11.51%) and Actinomycetota (8.16%); the dominant bacterial family was Prevotellaceae (43.20%), followed by Lachnospiraceae (8.86%). The genus *Prevotella* (42.80%) was predominant bacterial genera, followed by *g__unknown_d__Bacteria* (5.13%), *Ruminococcus* (2.93%), *Faecalibacterium* (2.47%), *Bifidobacterium* (2.16%) and *Bacteroides* (1.96%)*.*

### Comparison of the rumen microbial community composition, and diversity when cows were fed diets with differing CP concentrations

**Fig. 2:**
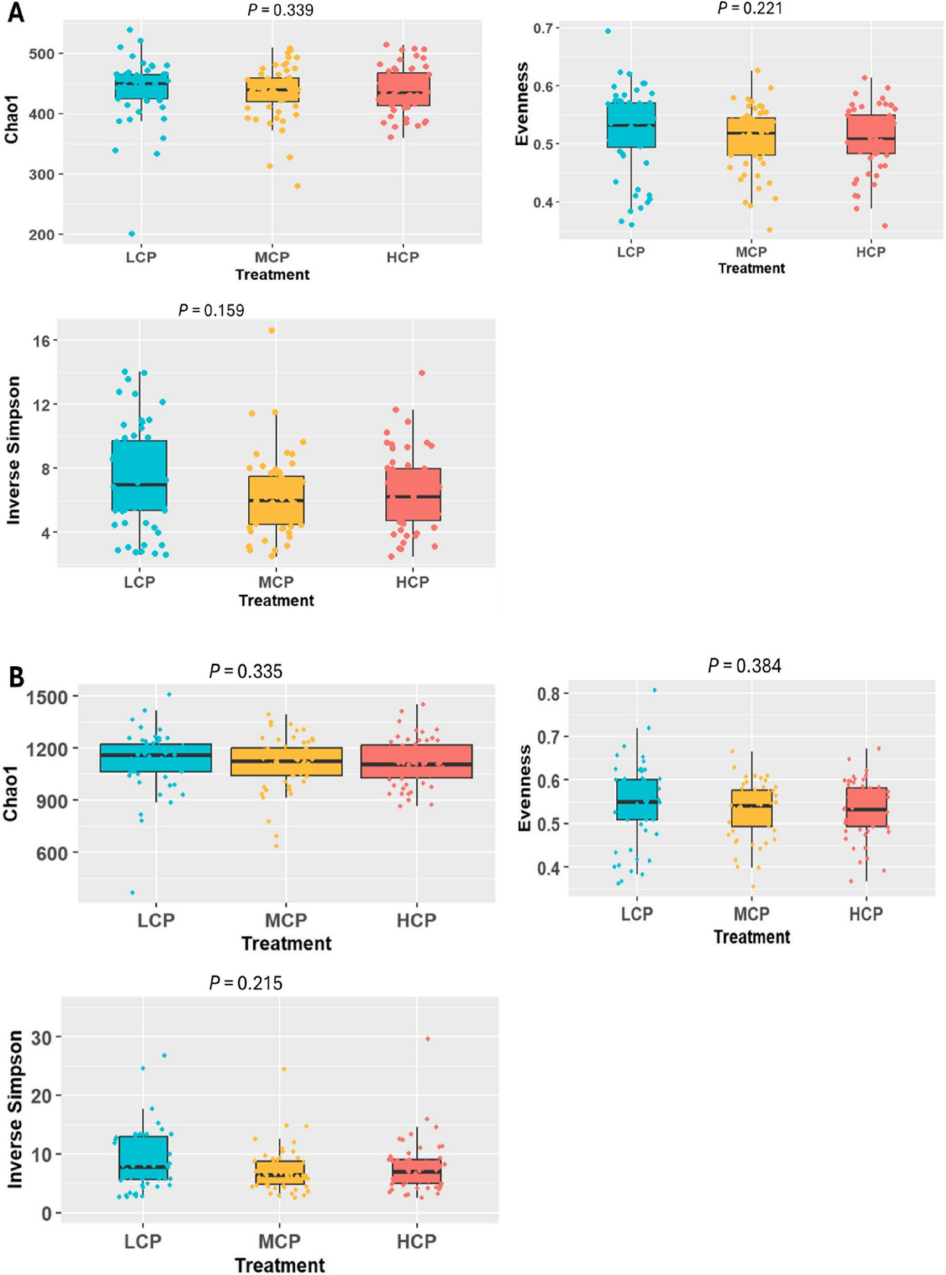
Alpha diversity indices of the rumen microbiota at the family level (**A**) and genus level (**B**) across diet CP concentrations (*n* = 177), Treatments: LCP (low CP; 15% CP), MCP (medium CP; 16% CP) and HCP (high CP; 17% CP)

**Fig. 3:**
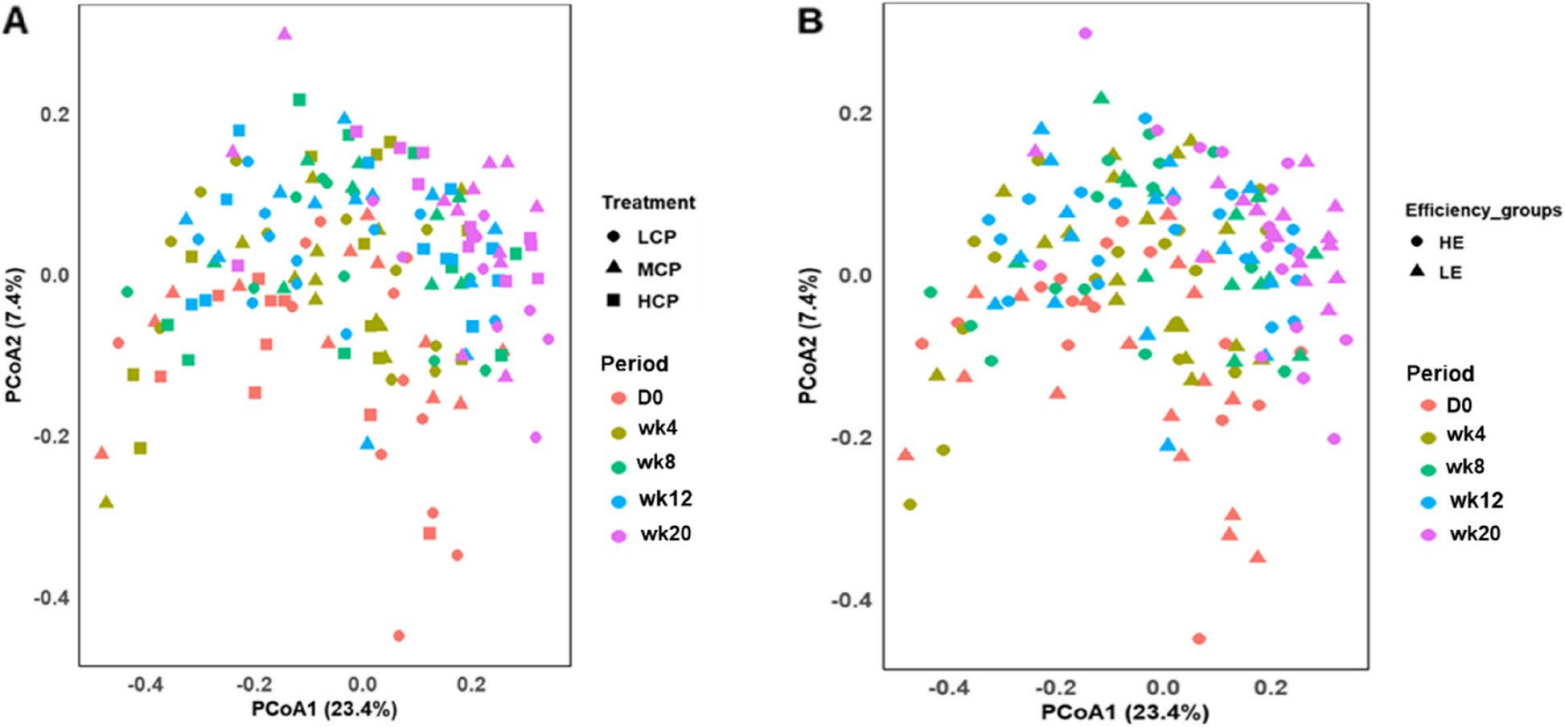
PCoA plot of the rumen microbial compositional profiles across diet (**A**) and efficiency groups (**B**) at the genus level (*n* = 177). The periods included D0 (baseline); wk4 (week 4); wk8 (week 8); wk12 (week 12); wk20 (week 20) and the treatments included LCP (low CP; 15% CP), MCP (medium CP; 16% CP) and HCP (high CP; 17% CP). HE, high efficiency; LE, low efficiency

We employed various alpha diversity indices, including the Chao1 index (richness), Pielou index (evenness) and Inverse Simpson diversity index to examine the impact of reducing diet CP concentrations from 17% to 15% on rumen microbial diversity across various taxonomic levels. At the phylum level, microbial richness, evenness and diversity remained unchanged in response to changes in diet CP concentrations (*P* > 0.05). Similarly, no significant effects on microbiota richness, evenness or diversity at the family or genus levels were detected (*P* > 0.05, Fig. 2A and B). Moreover, the beta diversity (PCoA) results did not show a distinct separation (Fig. S2A). Despite the first two PCoA axes covering 26.6% of the total variation in the data at the phylum level, PERMANOVA revealed no significant differences (*P* = 0.243, Table S3A). At the family level, 29.6% of the total variation was explained by the first two PCoA axes, however, PERMANOVA did not detect significant differences (*P* = 0.274, Table S3A). Likewise, at the genus level, PCoA1 and PCoA2 explained 23.4% and 7.4% of the total variation, respectively (Fig. 3A), with PERMANOVA again revealing no significant differences (*P* = 0.286, Table S3A**).** In addition, cows offered varying diet CP concentrations exhibited comparable differential abundances at the phylum, family and genus levels (*P* > 0.05). Full taxonomic data can be found in an additional file (see Additional file 2: Taxonomic and functional data).

### Comparison of the rumen microbial community composition, and diversity among efficiency groups

Microbiota richness and diversity were not significantly different between HE and LE cows at the phylum level (*P* > 0.05). However, we found a tendency for increased microbiota evenness in LE cows (*P* = 0.088). At the family level, no significant differences in microbiota richness, evenness and diversity were observed between HE and LE cows (*P* > 0.05). These results were consistent at the genus level (*P* > 0.05). Furthermore, PCoA showed no distinct clustering among HE and LE cows at various taxonomic levels. At the phylum level, although PCoA1 and PCoA2 accounted for 26.6% of the total variation (Fig. S2B), PERMANOVA results showed no significant differences among the two groups (*P* > 0.05, Table S3B). Similarly, at the family level, microbial composition among efficiency groups was statistically comparable, as indicated by PERMANOVA (*P* > 0. 05), with 21.9% and 7.7% of the variation explained by PCoA1 and PCoA2, respectively (Fig. S3B). At the genus level, PERMANOVA indicated that samples from HE cows showing a minimal separation compared to those from LE cows (Table S3B**,** Fig. 3B). Our analysis indicates that phylum abundance in HE cows was similar to that observed in LE cows (*P* > 0.05), these results were also consistent at the family and genus levels. However, when considering the effects of diet and time, f__Bacteroidaceae were abundant in the rumens of HE cows fed the HCP diet at week 8 (*P* < 0.05). Conversely, LE cows on the HCP diet exhibited greater abundances of f__Methanobacteriaceae and *g__Methanobrevibacter* during week 12 (*P* < 0.01).

### Natural fluctuations in rumen microbial community composition and diversity over the sampling periods

At the phylum level, diversity did not significantly change over the sampling period (*P* > 0.05). However, a lower richness was identified at week 20 compared to other weeks. Additionally, evenness was influenced by the sampling period (*P* = 0.008); evenness at baseline was comparable to that in all other periods but was substantially lower at weeks 4 and 8 compared to week 20. At the family level, neither diversity nor evenness varied significantly over the study period (*P* > 0.05). However, richness decreased at week 20 (*P* < 0.001). Likewise, we observed a reduction in richness at week 20 compared to all other periods (*P* < 0.001), and there was a tendency for increased evenness at week 20 (*P* = 0.069) at the genus level. However, diversity remained constant throughout the sampling periods (*P* > 0.05). Furthermore, PCoA plot indicated that samples collected at different periods, especially at week 20, exhibited moderate clustering at the phylum level (Fig. S2B), with 26.6% of the variation captured by PCoA1 and PCoA2 (PERMANOVA; *P* = 0.006, Table S3C). These results were consistent at the family level, with the first and second axes accounting for 29.6% of the total variation (PERMANOVA; *P* = 0.001, Table S3C). At the genus level, microbial composition was influenced by sampling period, as indicated by PERMANOVA (*P* = 0.001, Table S3C), with both PCoA1 and PCoA2 explaining 23.4% and 7.4% of the total variation, respectively (Fig. 3).

Phylum abundance was not affected at base line or at weeks 4, 8 or 12 (*P* > 0.05). In contrast, the abundance of p__Euryarchaeota, p__unknown_d__Bacteria*,* and p__Fibrobacterota was higher at week 20 (*P* < 0.01). At the family level, the following families were more abundant at baseline: Lachnospiraceae, Clostridiaceae, Staphylococcaceae, Lactobacillaceae and Methylobacteriaceae. During week 4, f__Bacillaceae, f__Succinivibrionaceae, f__Barnesiellaceae, and f__Muribaculaceae were differentially abundant (*P* < 0.05). During week 8, we observed the prevalence of f__Bacteroidaceae, f__unknown_o__Bacteroidales and f__Coprobacillaceae (*P* < 0.05). Moreover, f__Sphingobacteriaceae, f__Chitinophagaceae and f__Spirosomaceae*—*exhibited significant abundance at week 12. At week 20, several taxa families showed differential abundances (*P* < 0.01), including Methanobacteriaceae, Fibrobacteraceae, Desulfitobacteriaceae, Methanosarcinaceae, Eubacteriaceae, Acidaminococcaceae, Pseudoalteromonadaceae, and Veillonellaceae along with unknown families from domain Bacteria and phylum Bacillota and orders Eubacteriales and Enterobacterales. At the genus level, nine genera, including *Blautia, Butyrivibrio, Clostridium,* and *Pseudobutyrivibrio,* were significantly abundant at baseline (Table 3). The *g__Bacteroides, g__Sodaliphilus, g__Succinivibrio, g__Lactobacillus* and *g__unknown_p__Bacteroidota* had relatively high abundances at week 4 (*P* < 0.05, Table 3). Moreover, three genera, such as *Parabacteroides, Chitinophaga* and an unknown genus from the order Bacteroidales were more abundant at week 8 (*P* < 0.01). During week 12, *g__Hymenobacter, g__Microvirga, g__Sporomusa* and *g__Celeribacter* showed significant abundances. At week 20, various differentially abundant genera were detected (*P* < 0.05)*,* including *Dialister, Methanobrevibacter* and *Fibrobacter* (Table 3).

**Table 3:** Differentially abundant genera over the sampling periods

Genus	Sampling period^1^	LDA score	*P*-values
*g__Blautia*	D0	3.62	0.003
*g__Butyrivibrio*	D0	3.38	0.004
*g__Clostridium*	D0	3.20	0.017
*g__unknown_o__Eubacteriales*	D0	3.11	0.004
*g__Lachnoclostridium*	D0	3.06	0.004
*g__Staphylococcus*	D0	2.80	0.001
*g__Methylobacterium*	D0	2.09	0.009
*g__Saccharopolyspora*	D0	2.05	0.003
*g__Pseudarthrobacter*	D0	2.27	0.008
*g__Bacteroides*	Week 4	3.51	0.001
*g__Intestinibaculum*	Week 4	3.47	0.012
*g__Sodaliphilus*	Week 4	3.43	0.019
*g__Parafannyhessea*	Week 4	3.02	0.004
*g__unknown_p__Bacteroidota*	Week 4	2.65	0.031
*g__Succinivibrio*	Week 4	2.28	0.002
*g__Lactobacillus*	Week 4	2.09	0.020
*g__unknown_o__Bacteroidales*	Week 8	2.97	0.001
*g__Parabacteroides*	Week 8	2.34	0.023
*g__Chitinophaga*	Week 8	2.03	0.002
*g__Hymenobacter*	Week 12	2.58	0.023
*g__Microvirga*	Week 12	1.89	0.028
*g__Sporomusa*	Week 12	1.56	0.015
*g__Celeribacter*	Week 12	1.47	0.012
*g__Methanobrevibacter*	Week 20	3.94	< 0.001
*g__unknown_d__Bacteria*	Week 20	3.76	0.013
*g__Fibrobacter*	Week 20	3.54	0.001
*g__unknown_f__Lachnospiraceae*	Week 20	3.14	0.021
*g__Phascolarctobacterium*	Week 20	2.88	0.003
*g__unknown_p__Bacillota*	Week 20	2.88	0.016
*g__Dialister*	Week 20	2.76	< 0.001
*g__Acidaminococcus*	Week 20	2.73	0.001
*g__Methanosphaera*	Week 20	2.71	< 0.001
*g__unknown_f__Oscillospiraceae*	Week 20	2.61	0.041
*g__unknown_o__Enterobacterales*	Week 20	2.54	0.025
*g__Methanosarcina*	Week 20	2.08	0.002
*g__Pseudoalteromonas*	Week 20	2.12	0.019

^1^Sampling period: D0 (baseline), week 4, week 8, week 12 and week 20

### Differential functional profiles of the rumen microbiome across different diet CP concentrations

**Fig. 4:**
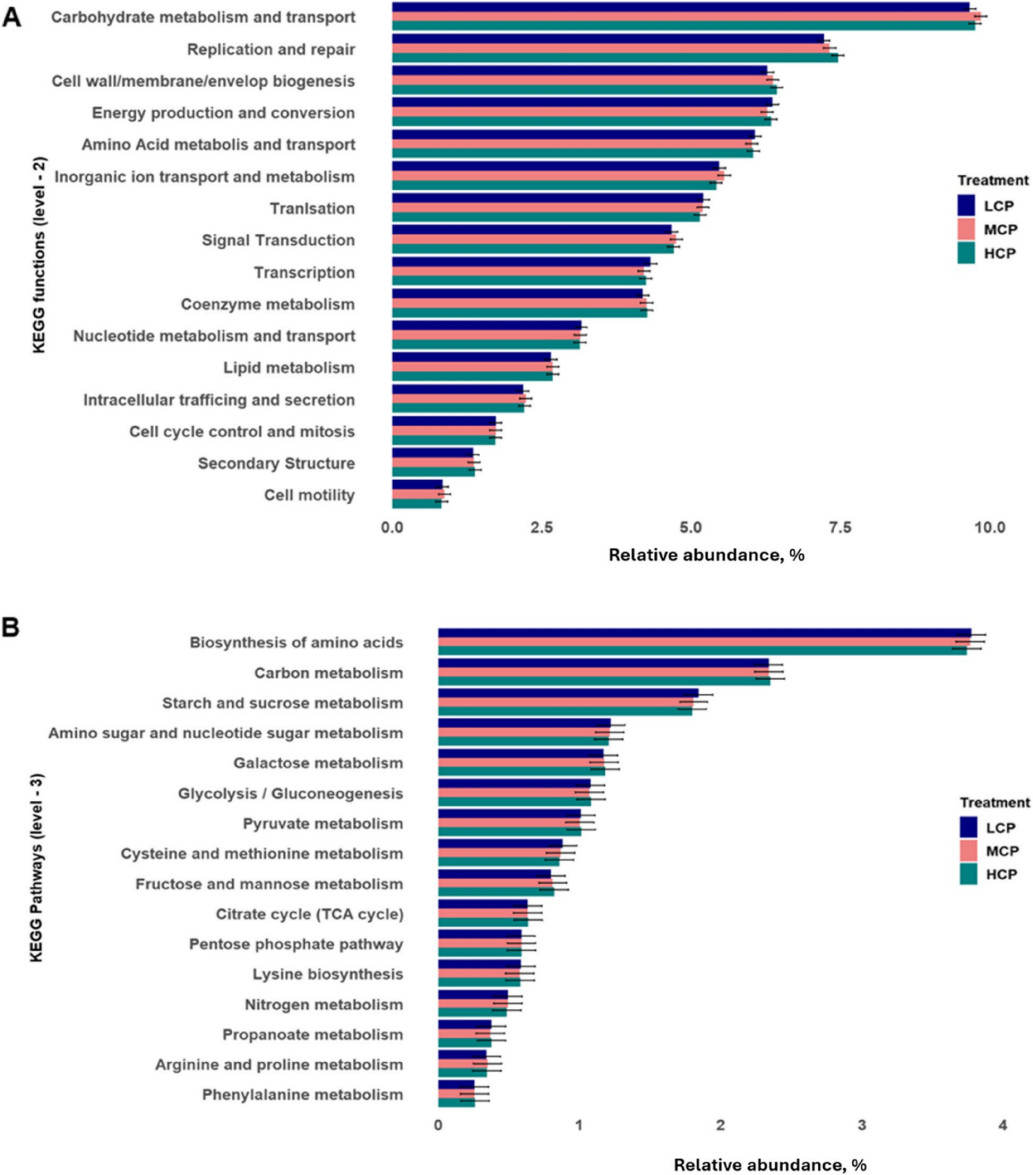
The relative abundance of KEGG functions (**A**; level – 2) and KEGG pathways (**B**; level – 3) across different CP concentrations (*n* = 177). No significantly different pathways (levels 2 and 3) were observed between different diet CP concentrations (*P *> 0.05)

For functional analysis of the rumen microbiome, we considered KEGG pathways, KEGG modules, KEGG enzymes and genes encoding CAZymes. For the KEGG pathway profiles, 419 level-3 pathways and 24 level-2 pathway functional categories (Clusters of Orthologous Groups; COGs) were considered for downstream analysis. No significant differences were observed in either second-level categories (Fig. 4A) or third-level pathways (Fig. 4B) between cows fed diets with varying CP concentrations (*P* > 0.05). Likewise, module abundance was not substantially influenced by diet CP levels (*P* > 0.05). For KEGG enzyme analysis, a total of 2,651 genes encoding EC (Enzyme Commission) numbers were identified and analysed. We found a comparable abundance of microbial enzymes in cows offered different CP levels (*P* > 0.05). The raw functional data is presented in an additional file (see Additional file 2: Taxonomic and functional data).

For the CAZyme profiles, we identified a total of 113 genes encoding CAZymes, which are grouped into different families: 53 glycoside hydrolases (GHs), 46 glycosyl transferases (GTs), seven carbohydrate-binding modules (CBMs), four polysaccharide lyases (PLs), two carbohydrate esterases (CEs), and one auxiliary activity (AA). The most abundant CAZymes in this study were GH13 (13.64%), followed by GH3 (9.81%), GH31 (7.74%), CBM48 (6.14%), GT51 (4.43%), GT2 (4.30%) and GH77 (4.27%). Our analysis revealed that CAZyme abundance was comparable among cows receiving diets with varying CP concentrations (*P* > 0.05).

### Comparative functional profiles of the rumen microbiome between HE and LE cows

**Fig. 5:**
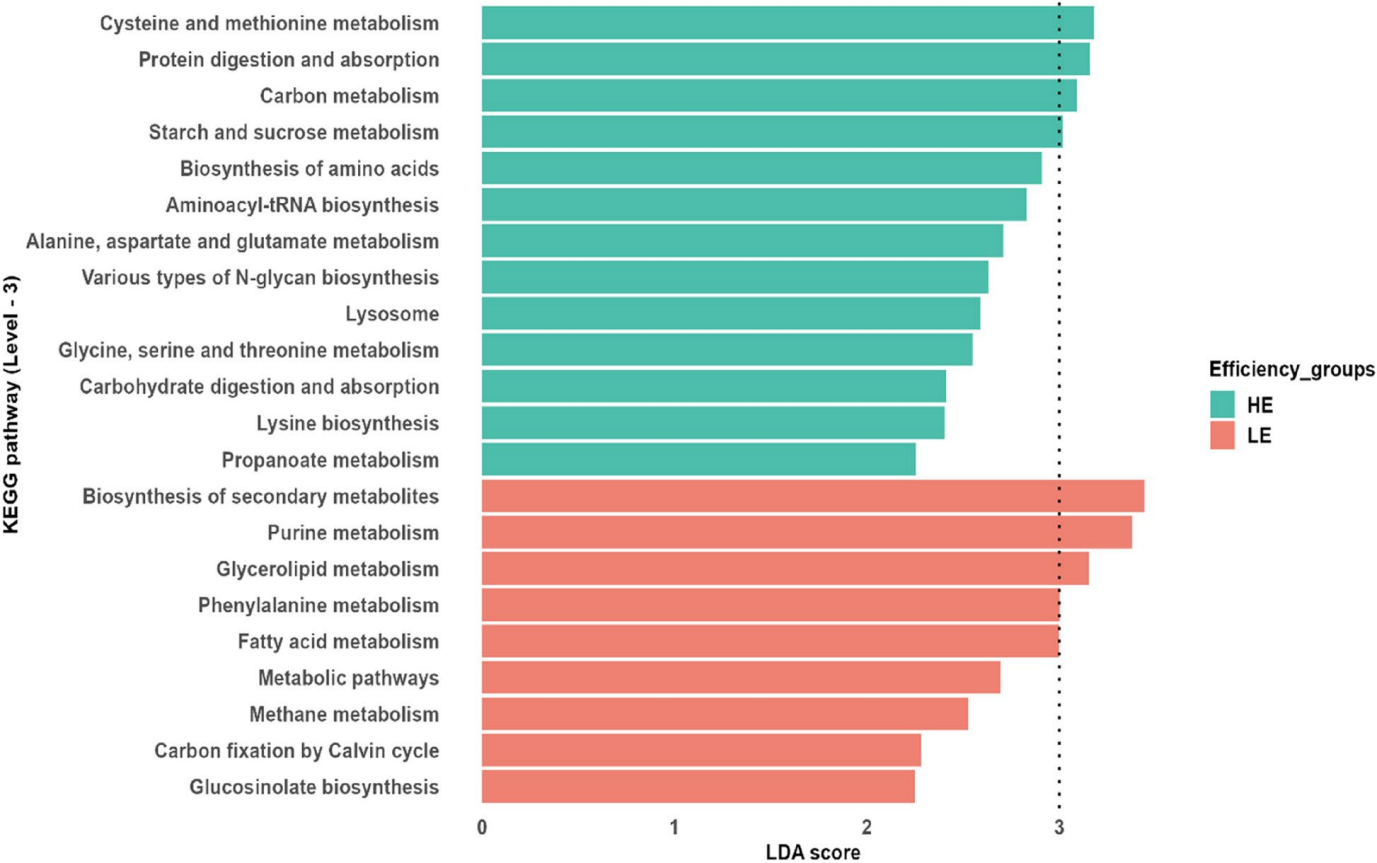
Differentially abundant KEGG pathways among HE and LE cows across different CP concentrations. Significant differences were tested by LEfSe analysis (*P *< 0.05, LDA score > 2, *n* = 177), with dotted lines indicating highly significant pathways among efficiency groups with LDA scores greater than 3

In terms of RFI groups, the second-level functional categories and third-level KEGG pathways in HE cows were comparable to those in LE cows (*P* < 0.05). However, we identified significant differential abundances among HE and LE cows for various functional parameters within the same diet and time context. During week 4, ko00300 (lysine biosynthesis) in the MCP, ko04070 (phosphatidylinositol signaling system), ko00970 (aminoacyl-tRNA biosynthesis), and ko00500 (starch and sucrose metabolism) in the HCP, and ko00250 (alanine, aspartate and glutamate metabolism) in the LCP diet were enriched in HE cows (*P* < 0.05, Fig. 5). On the contrary, ko00230 (purine metabolism) was abundant in LE cows offered the HCP diet (*P* < 0.05). During week 8, ko04974 (protein digestion and absorption), ko01200 (carbon metabolism) and ko03018 (RNA degradation) in HE cows offered LCP, and ko00640 (propanoate metabolism) in HE cows receiving HCP diet were highly abundant (*P* < 0.05), whilst LE cows in the HCP and MCP diets exhibited upregulation of ko00360 (phenylalanine metabolism) and ko01110 (biosynthesis of secondary metabolites), respectively (*P* < 0.05, Fig. 5).

Moreover, at week 12 the ko04973 (carbohydrate digestion and absorption), ko00513 (various types of N-glycan biosynthesis), and ko04142 (lysosome) in HE cows on the HCP and ko01230 (biosynthesis of amino acids) in HE cows fed the LCP diet showed greater abundances (*P* < 0.05), whereas ko01212 (fatty acid metabolism), and ko00680 (methane metabolism) in the HCP and ko00710 (carbon fixation by Calvin cycle) in the LCP were upregulated in LE cows (*P* < 0.05). At week 20, ko00400 (phenylalanine, tyrosine and tryptophan biosynthesis), and ko00260 (glycine, serine and threonine metabolism) in the LCP diet, ko00270 (cysteine and methionine metabolism) in the HCP diet and ko04142 (lysosome) in the MCP diet were significantly more abundant in HE cows, while both ko00966 (glucosinolate biosynthesis) and ko00561 (glycerolipid metabolism) in the LCP diet and ko01100 (metabolic pathways) in the HCP diet were more abundant in LE cows (*P* < 0.05, Fig. 5).

Although the abundance of KEGG modules did not differ between HE and LE cows (*P* > 0.05) regardless of diet or sampling period, we identified several modules significant between the two groups when considering diet and temporal effects. The M00360 (aminoacyl-tRNA biosynthesis, prokaryotes), M00359 (aminoacyl-tRNA biosynthesis, eukaryotes), M00228 (putative glutamine transport system), M00232 (general L-amino acid transport system) and M00275 (phosphotransferase system, cellobiose-specific II component) in the HCP diet were higher in HE cows at week 4 (*P* < 0.05), while M00135 (GABA biosynthesis, eukaryotes, putrescine → GABA) in the MCP was more abundant in LE cows offered MCP (*P* < 0.05). At week 8 M00375 (hydroxypropionate-hydroxybutylate cycle) in the MCP, M00554 (nucleotide sugar biosynthesis, galactose → UDP-galactose), and M00115 (NAD biosynthesis, aspartate → quinolinate → NAD) in the HCP and M00362 (nucleotide sugar biosynthesis, prokaryotes) and M00335 (secretion system) in the LCP were upregulated in HE cows (*P* < 0.05), in contrast, four modules M00742 (aminoglycoside resistance, protease FtsH), M00039 (monolignol biosynthesis, phenylalanine/tyrosine → monolignol), M00350 (capsaicin biosynthesis, L-Phenylalanine → Capsaicin) and M00240 (iron complex transport system) in the HCP were enriched in LE cows.

During week 12, M00072 (n-glycosylation by oligosaccharyltransferase), M00117 (ubiquinone biosynthesis, prokaryotes, chorismate (+ polyprenyl-PP) → ubiquinol), M00535 (isoleucine biosynthesis, pyruvate → 2-oxobutanoate) and M00220 (COPII complex [Coat Protein Complex II]) in the HCP diet and M00549 (nucleotide sugar biosynthesis, glucose → UDP-glucose) and M00343 (archaeal proteasome) in the LCP diet were abundant in HE cows, whereas M00122 (cobalamin biosynthesis, cobinamide → cobalamin), M00836 (coenzyme F430 biosynthesis, sirohydrochlorin → coenzyme F430), and M00130 (inositol phosphate metabolism, PI → PIP2 → Ins [1,4,5] P3 → Ins [1,3,4,5] P4) in the HCP diet were greater in LE cows (*P* < 0.05). Finally, M00580 (pentose phosphate pathway, archaea, fructose 6P → ribose 5P), M00288 (replication protein A complex) and M00264 (DNA polymerase II complex, archaea) in the LCP diet and M00184 (RNA polymerase, archaea) and M00546 (purine degradation, xanthine → urea) in the MCP diet were enriched in HE cows at week 20, whereas M00052 (pyrimidine ribonucleotide biosynthesis, UMP → UDP/UTP,CDP/CTP) and M00023 (Tryptophan biosynthesis, chorismate → tryptophan) in the HCP, M00694 (pyrimidine ribonucleotide biosynthesis, UMP → UDP/UTP,CDP/CTP) in the LCP and M00483 (NreB-NreC [dissimilatory nitrate/nitrite reduction] two-component regulatory system) in the MCP diet were all enriched in LE cows (*P* < 0.05).

No differences were observed in the abundance of genes encoding KEGG enzymes in the rumens of HE and LE cows (*P* > 0.05). However, distinct variations in gene abundance were noted between the two groups of cows fed the same diet during the same period. Specifically, in the biosynthesis of amino acids pathway, EC 4.2.1.33 (3-isopropylmalate dehydratase) and EC 4.2.1.35 (R-2-methylmalate dehydratase) were enriched in HE cows, while the genes EC 4.1.1.48 (indole-3-glycerol-phosphate synthase) and EC 2.4.2.18 (anthranilate phosphoribosyltransferase) were more abundant in LE cows (*P* < 0.05). Within the cysteine and methionine metabolism pathway, two genes EC 2.3.1.31 (homoserine O-acetyltransferase) and EC 3.3.1.1 (adenosylhomocysteinase) were higher in HE cows, while EC 2.1.1.14 (5-methyltetrahydropteroyltriglutamat-homocysteine S-methyltransferase) exhibited enrichment in LE cows (*P* < 0.01). Three genes involved in the purine metabolism pathway EC 6.3.5.2 (GMP synthase [glutamine-hydrolysing]), EC 2.4.2.7 (adenine phosphoribosyltransferase) and EC 3.5.4.2 (adenine deaminase) and one gene involved in phenylalanine metabolism EC 4.3.1.24 (phenylalanine ammonia-lyase) were significantly enriched in LE cows (*P* < 0.05). Additionally, EC 1.2.1.18 (malonate-semialdehyde dehydrogenase (acetylating)) and EC 1.2.1.27 (methylmalonate-semialdehyde dehydrogenase (CoA-acylating)) associated with propanoate metabolism pathway were more abundant in HE cows (*P* < 0.05). In the glycerolipid metabolism pathway, two genes EC 2.7.1.165 (glycerate 2-kinase) and EC 2.7.7.9 (UTP-glucose-1-phosphate uridylyltransferase) were notably abundant in LE cows (*P* < 0.05). Furthermore, both HE and LE cows exhibited similar CAZyme gene abundance (*P* > 0.05). But with the same diet and time, both GH77 in the LCP diet at week 20 and GT66 in the HCP diet at week 12 were more abundant in HE cows, while GT4 in the HCP diet at week 8 and GH59 in the HCP diet at week 20 were more prevalent in LE cows (*P* < 0.05).

### Natural fluctuations in functional profiles of the rumen microbiome across sampling periods

We found 12 significantly abundant second-level functional categories (*P* < 0.05). Among them, “function unknown”, “transcription” and “extracellular structures” were abundant at baseline, “defense mechanisms”, “cell wall/membrane/envelope biogenesis”, and “coenzyme transport and metabolism” were enriched at weeks 4, 8 and 12, respectively, and six COGs, such as “amino acid transport and metabolism”, “energy production and conversion”, “signal transduction mechanisms”, “lipid transport and metabolism”, “secondary metabolite biosynthesis”, “transport and catabolism” and “extracellular structures” were more abundant at week 20. For pathway analysis, we found nine differentially abundant pathways at baseline (*P* < 0.01; Table 4), while ten pathways were more abundant at week 4 (Table 4). At week 8, ko00061 (fatty acid biosynthesis), ko00540 (lipopolysaccharide biosynthesis), and ko04974 (protein digestion and absorption) were more abundant (*P* < 0.01). During week 12, we observed a higher abundance of ko01212 (fatty acid metabolism), ko00740 (metabolism of cofactors and vitamins), and ko00510 (N-Glycan biosynthesis). The rumen samples collected at week 20 showed upregulation of ko00680 (methane metabolism), ko01200 (carbon metabolism), ko00720 (other carbon fixation pathways), ko01230 (biosynthesis of amino acids), ko00400 (phenylalanine, tyrosine and tryptophan biosynthesis), ko01110 (biosynthesis of secondary metabolites), ko00966 (glucosinolate biosynthesis) and ko01210 (2-Oxocarboxylic acid metabolism) (*P* < 0.01).

**Table 4:** Differentially abundant KEGG pathways over the sampling periods

KEGG entry	Name	Pathway	Period^1^	LDA score	*P*_adj_
ko00240	Pyrimidine metabolism	Nucleotide metabolism	D0	2.65	< 0.001
ko03030	DNA replication	Replication and repair	D0	2.56	< 0.001
ko00230	Purine metabolism	Nucleotide metabolism	D0	2.55	< 0.001
ko00520	Amino sugar and nucleotide sugar metabolism	Carbohydrate metabolism	D0	2.21	< 0.001
ko04142	Lysosome	Cellular Processes; Transport and catabolism	D0	2.10	< 0.001
ko00603	Glycosphingolipid biosynthesis—globo and isoglobo series	Glycan biosynthesis and metabolism	D0	2.08	0.002
ko00531	Glycosaminoglycan degradation	Glycan biosynthesis and metabolism	D0	2.08	< 0.001
ko02060	Phosphotransferase system (PTS)	Environmental Information Processing; Membrane transport	D0	2.07	0.007
ko00604	Glycosphingolipid biosynthesis—ganglio series	Glycan biosynthesis and metabolism	D0	2.05	< 0.001
ko00250	Alanine, aspartate and glutamate metabolism	Amino acid metabolism	Week 4	2.32	< 0.001
ko03010	Ribosome	Genetic Information Processing; Translation	Week 4	2.24	0.008
ko00970	Aminoacyl-tRNA biosynthesis	Genetic Information Processing; Translation	Week 4	2.20	0.022
ko00460	Cyanoamino acid metabolism	Other amino acid metabolism	Week 4	2.17	0.011
ko00940	Phenylpropanoid biosynthesis	Biosynthesis of other secondary metabolites	Week 4	2.16	0.017
ko03018	RNA degradation	Genetic Information Processing; Folding, sorting and degradation	Week 4	2.15	0.001
ko00910	Nitrogen metabolism	Energy metabolism	Week 4	2.10	0.001
ko00010	Glycolysis/Gluconeogenesis	Carbohydrate metabolism	Week 4	2.10	0.001
ko00051	Fructose and mannose metabolism	Carbohydrate metabolism	Week 4	2.06	0.027
ko00190	Oxidative phosphorylation	Energy metabolism	Week 4	2.01	0.023
ko00061	Fatty acid biosynthesis	Lipid metabolism	Week 8	2.20	0.005
ko00540	Lipopolysaccharide biosynthesis	Glycan biosynthesis and metabolism	Week 8	2.15	0.048
ko04974	Protein digestion and absorption	Organismal Systems; Digestive system	Week 8	3.48	0.020
ko01212	Fatty acid metabolism	Lipid metabolism	Week 12	2.11	0.017
ko00740	Metabolism of cofactors and vitamins	Metabolism of cofactors and vitamins	Week 12	1.83	0.004
ko00510	N-Glycan biosynthesis	Glycan biosynthesis and metabolism	Week 12	1.45	< 0.001
ko01110	Biosynthesis of secondary metabolites	Biosynthesis of secondary metabolites	Week 20	2.59	0.015
ko00680	Methane metabolism	Energy metabolism	Week 20	2.51	< 0.001
ko01230	Biosynthesis of amino acids	Aminoacid biosynthesis and metabolism	Week 20	2.51	0.001
ko01200	Carbon metabolism	Energy metabolism	Week 20	2.51	0.001
ko00400	Phenylalanine, tyrosine and tryptophan biosynthesis	Aminoacid metabolism	Week 20	2.36	< 0.001
ko00966	Glucosinolate biosynthesis	Biosynthesis of other secondary metabolites	Week 20	2.90	< 0.001
ko01210	2-Oxocarboxylic acid metabolism	Energy metabolism	Week 20	2.18	0.002
ko00720	Other carbon fixation pathways	Energy metabolism	Week 20	2.14	0.030

^1^Sampling period: D0 (baseline), week 4, week 8, week 12 and week 20

For module analysis, 18 modules were upregulated at baseline (*P* < 0.01; Table 5). At week 4, 12 modules, such as M00135 (GABA biosynthesis, eukaryotes, putrescine → GABA), M00045 (histidine degradation, histidine → N-formiminoglutamate → glutamate), M00009 (Citrate cycle [TCA cycle, Krebs cycle]), M00114 (ascorbate biosynthesis, plants, fructose-6P → ascorbate), M00014 (glucuronate pathway [uronate pathway]), M00308 (semi-phosphorylative Entner-Doudoroff pathway, gluconate → glycerate-3P), M00359 (aminoacyl-tRNA biosynthesis, eukaryotes), M00360 (aminoacyl-tRNA biosynthesis, prokaryotes), M00335 (secretion system), M00529 (denitrification, nitrate → nitrogen), M00178 (ribosome, bacteria) and M00144 (NADH:quinone oxidoreductase, prokaryotes) were more abundant (*P* < 0.01). During week 8, the following modules were differentially abundant: M00060 (KDO2-lipid A biosynthesis, Raetz pathway, LpxL-LpxM type), M00063 (CMP-KDO biosynthesis), M00080 (lipopolysaccharide biosynthesis, inner core → outer core → O-antigen), M00258 (Putative ABC transport system), M00222 (Phosphate transport system), M00255 (lipoprotein-releasing system), M00086 (beta-Oxidation, acyl-CoA synthesis), M00579 (phosphate acetyltransferase-acetate kinase pathway, acetyl-CoA → acetate), and M00129 (ascorbate biosynthesis, animals, glucose-1P → ascorbate). Additionally, microbes during week 12 exhibited a significant abundance of M00125 (riboflavin biosynthesis, plants and bacteria, GTP → riboflavin/FMN/FAD), M00026 (histidine biosynthesis, PRPP → histidine), M00535 (Isoleucine biosynthesis, pyruvate → 2-oxobutanoate), M00024 (phenylalanine biosynthesis, chorismate → phenylpyruvate → phenylalanine), M00620 (incomplete reductive citrate cycle, acetyl-CoA → oxoglutarate), M00356 (methanogenesis, methanol → methane), M00083 (fatty acid biosynthesis, elongation), and M00177 (ribosome, eukaryotes). Finally, 18 modules showed an increased abundance at week 20 (*P* < 0.01; Table 5).

**Table 5:** Differentially abundant KEGG modules at baseline (D0) and week 20

KEGG module	Name	Pathway	Period	LDA score	*P*_adj_
M00079	Keratan sulfate degradation	Glycan metabolism; Glycosaminoglycan metabolism	D0	2.90	< 0.001
M00429	Competence-related DNA transformation transporter	Environmental information processing	D0	2.83	< 0.001
M00035	Methionine degradation	Amino acid metabolism; Cysteine and methionine metabolism	D0	2.80	< 0.001
M00565	Trehalose biosynthesis, D-glucose 1P → trehalose	Carbohydrate metabolism; Other carbohydrate metabolism	D0	2.78	0.029
M00081	Pectin degradation	Carbohydrate metabolism; Other carbohydrate metabolism	D0	2.57	0.037
M00016	Lysine biosynthesis, succinyl-DAP pathway, aspartate → lysine	amino acid metabolism	D0	2.56	0.004
M00049	Adenine ribonucleotide biosynthesis, IMP → ADP,ATP	Nucleotide metabolism; Purine metabolism	D0	2.55	< 0.001
M00276	PTS system, mannose-specific II component	Environmental information processing	D0	2.42	0.002
M00280	PTS system, glucitol/sorbitol-specific II component	Environmental information processing	D0	2.41	< 0.001
M00172	C4-dicarboxylic acid cycle, NADP—malic enzyme type	Energy metabolism; Carbon fixation	D0	2.40	0.002
M00169	CAM (Crassulacean acid metabolism), light	Energy metabolism; Carbon fixation	D0	2.40	0.004
M00020	Serine biosynthesis, glycerate-3P → serine	Amino acid metabolism; Serine and threonine metabolism	D0	2.33	0.001
M00550	Ascorbate degradation, ascorbate → D-xylulose-5P	Carbohydrate metabolism; Other carbohydrate metabolism	D0	2.31	0.006
M00046	Pyrimidine degradation, uracil → beta-alanine, thymine → 3-aminoisobutanoate	Nucleotide metabolism; Pyrimidine metabolism	D0	2.26	0.001
M00005	PRPP biosynthesis, ribose 5P → PRPP	Carbohydrate metabolism; Central carbohydrate metabolism	D0	2.26	0.002
M00008	Entner-Doudoroff pathway, glucose-6P → glyceraldehyde-3P + pyruvate	Carbohydrate metabolism; Central carbohydrate metabolism	D0	2.18	< 0.001
M00283	PTS system, ascorbate-specific II component	Environmental information processing	D0	2.11	< 0.001
M00006	Pentose phosphate pathway, oxidative phase, glucose 6P → ribulose 5P	Carbohydrate metabolism; Central carbohydrate metabolism	D0	2.06	0.001
M00023	Tryptophan biosynthesis, chorismate → tryptophan	Nucleotide and amino acid metabolism	Week 20	3.06	< 0.001
M00567	Methanogenesis, CO_2_ → methane	Energy metabolism	Week 20	2.84	< 0.001
M00570	Isoleucine biosynthesis, threonine → 2-oxobutanoate → isoleucine	Amino acid metabolism; Branched-chain amino acid metabolism	Week 20	2.58	0.027
M00357	Methanogenesis, acetate → methane	Energy metabolism; Methane metabolism	Week 20	2.49	0.001
M00432	Leucine biosynthesis, 2-oxoisovalerate → 2-oxoisocaproate	Amino acid metabolism; Branched-chain amino acid metabolism	Week 20	2.40	< 0.001
M00546	Purine degradation, xanthine → urea	Nucleotide and amino acid metabolism	Week 20	2.34	0.029
M00022	Shikimate pathway, phosphoenolpyruvate + erythrose-4P → chorismate	Amino acid metabolism; Aromatic amino acid metabolism	Week 20	2.28	0.010
M00098	Acylglycerol degradation	Lipid metabolism; Lipid metabolism	Week 20	2.25	0.009
M00506	CheA-CheYBV (chemotaxis) two-component regulatory system	Environmental information processing	Week 20	2.25	0.001
M00596	Dissimilatory sulfate reduction, sulfate → H2S	Energy metabolism	Week 20	2.21	0.023
M00345	Formaldehyde assimilation, ribulose monophosphate pathway	Energy metabolism; Methane metabolism	Week 20	2.18	0.032
M00175	Nitrogen fixation, nitrogen → ammonia	Energy metabolism; Nitrogen metabolism	Week 20	2.16	0.002
M00483	NreB-NreC (dissimilatory nitrate/nitrite reduction) two-component regulatory system	Environmental information processing	Week 20	2.13	< 0.001
M00072	N-glycosylation by oligosaccharyltransferase	Glycan metabolism; Glycan biosynthesis	Week 20	2.07	< 0.001
M00608	2-Oxocarboxylic acid chain extension, 2-oxoglutarate → 2-oxoadipate → 2-oxopimelate → 2-oxosuberate	Energy metabolism; Methane metabolism	Week 20	2.03	< 0.001

In the analysis of KEGG enzymes, 25 significantly abundant enzymes were detected at baseline (*P* < 0.01; Table 6). At week 4, we found 18 significantly different enzymes, which are grouped into several metabolic pathways: carbohydrate metabolism: EC 3.2.1.4 (cellulase), EC 3.2.1.3 (glucan 1,4-alpha-glucosidase), EC 3.2.1.20 (alpha-glucosidase), EC 3.2.1.80 (fructan beta-fructosidase), EC 2.4.1.11 (glycogen(starch) synthase), EC 2.7.1.40 (pyruvate kinase), EC 2.7.7.13 (mannose-1-phosphate guanylyltransferase), EC 1.1.1.22 (UDP-glucose 6-dehydrogenase), EC 2.3.1.54 (formate C-acetyltransferase), EC 3.2.1.21 (beta-glucosidase) and EC 5.4.99.9 (UDP-galactopyranose mutase), energy metabolism: EC 1.6.5.3 (NADH:ubiquinone reductase [H^+^-translocating]), EC 1.4.1.13 (glutamate synthase [NADPH]), EC 1.4.1.14 (glutamate synthase [NADH], and EC 1.4.7.1 (glutamate synthase [ferredoxin]), amino acid metabolism: EC 1.4.1.13 (glutamate synthase [NADPH]), EC 1.4.1.14 (glutamate synthase [NADH]), and EC 3.2.1.21 (beta-glucosidase), secondary metabolite biosynthesis: EC 1.2.7.3 (2-oxoglutarate synthase), EC 1.2.7.11 (2-oxoacid oxidoreductase [ferredoxin]), EC 3.2.1.20 (alpha-glucosidase), EC 2.4.1.11 (glycogen(starch) synthase), EC 2.7.7.13 (mannose-1-phosphate guanylyltransferase) and EC 5.1.3.3 (Aldose 1-epimerase). At week 8, nine genes encoding enzymes were substantially abundant, such as EC 3.2.1.51 (alpha-L-fucosidase), EC 6.2.1.3 (long-chain-fatty-acid-CoA ligase), EC 2.4.2.14 (amidophosphoribosyltransferase), EC 5.3.1.12 (glucuronate isomerase), EC 2.7.1.2 (glucokinase), EC 1.4.4.2 (glycine dehydrogenase [aminomethyl-transferring]), EC 1.7.5.1 (nitrate reductase [quinone]), EC 1.4.3.16 (L-aspartate oxidase) and EC 1.4.1.4 (glutamate dehydrogenase [NADP^+^]). During week 12, several enzymes related to amino acid metabolism: EC 6.3.5.5 (carbamoyl-phosphate synthase [glutamine-hydrolysing]), EC 5.4.2.12 (phosphoglycerate mutase [2,3-diphosphoglycerate-independent]), EC 6.3.2.13 (UDP-N-acetylmuramoyl-L-alanyl-D-glutamate-2,6-diaminopimelate ligase), and EC 2.3.1.182 (R-citramalate synthase), glycan biosynthesis and metabolism: EC 2.7.8.43 (lipid A phosphoethanolamine transferase), and EC 3.5.1.108 (UDP-3-O-acyl-N-acetylglucosamine deacetylase), and EC 2.4.99.13, secondary metabolite biosynthesis: EC 3.1.3.5 (5'-nucleotidase) and EC 2.7.7.4 (sulfate adenylyltransferase), metabolism of cofactors and fatty acid biosynthesis: EC 2.3.1.41 (beta-ketoacyl-[acyl-carrier-protein] synthase I) and EC 4.2.1.59 (3-hydroxyacyl-[acyl-carrier-protein] dehydratase) and energy metabolism: EC 1.8.7.3 (ferredoxin:CoB-CoM heterodisulfide reductase) were detected. Moreover, at week 20, we identified 22 differentially abundant genes encoding KEGG enzymes (*P* < 0.01; Table 6).

**Table 6:** Differentially abundant KEGG enzymes at baseline (D0) and week 20

EC	Name	Class	Pathway	Period	LDA score	*P*_adj_
EC 3.2.1.52	Beta-N-acetylhexosaminidase	Hydrolases	Glycosaminoglycan degradation	D0	2.88	< 0.001
EC 5.1.3.2	UDP-glucose 4-epimerase	Isomerases	glycan biosynthesis and metabolism	D0	2.17	< 0.001
EC 3.2.1.96	Mannosyl-glycoprotein endo-beta-N-acetylglucosaminidase	Hydrolases	Other glycan degradation	D0	2.05	< 0.001
EC 2.7.1.40	Pyruvate kinase	Transferases	Pyruvate metabolism/Glycolysis/Gluconeogenesis	D0	2.03	0.018
EC 2.7.1.191	Protein-Npi-phosphohistidine-D-mannose phosphotransferase	Transferases	Fructose and mannose metabolism	D0	2.00	0.002
EC 3.2.1.82	Exo-poly-alpha-digalacturonosidase	Hydrolases	pectin degradation I	D0	2.44	0.001
EC 3.2.1.10	Oligo-1,6-glucosidase	Hydrolases	Galactose metabolism/Starch and sucrose metabolism	D0	2.38	0.002
EC 2.7.1.198	Protein-Npi-phosphohistidine-D-sorbitol phosphotransferase	Transferases	Fructose and mannose metabolism	D0	2.20	< 0.001
EC 2.4.1.20	Cellobiose phosphorylase	Transferases	Starch and sucrose metabolism	D0	2.22	0.004
EC 2.7.9.1	Pyruvate, phosphate dikinase	Transferases	Glycolysis/Gluconeogenesis	D0	2.35	0.001
EC 5.1.3.2	UDP-glucose 4-epimerase	Isomerases	Galactose metabolism	D0	2.17	< 0.001
EC 2.7.6.1	Ribose-phosphate diphosphokinase	Transferases	Pentose phosphate pathway	D0	2.19	0.005
EC 3.2.1.26	Beta-fructofuranosidase	Hydrolases	Galactose metabolism, Starch and sucrose metabolism	D0	2.42	0.037
EC 3.6.4.12	DNA helicase	Hydrolases	Replication and repair	D0	3.38	< 0.001
EC 6.5.1.2	DNA ligase (NAD^+^)	Ligases	Replication and repair	D0	2.30	< 0.001
EC 2.7.1.48	Uridine/cytidine kinase	Transferases	Pyrimidine metabolism	D0	2.15	0.038
EC 1.3.1.1	Dihydropyrimidine dehydrogenase (NAD^+^)	Oxidoreductases	Pyrimidine metabolism	D0	2.19	< 0.001
EC 6.3.4.4	Adenylosuccinate synthase	Ligases	Alanine, aspartate and glutamate metabolism	D0	2.20	0.003
EC 2.3.1.15	Glycerol-3-phosphate 1-O-acyltransferase	Transferases	Glycerolipid metabolism	D0	2.19	0.006
EC 1.2.1.10	Acetaldehyde dehydrogenase (acetylating)	Oxidoreductases	Phenylalanine metabolism	D0	2.19	0.020
EC 2.7.1.107	Diacylglycerol kinase (ATP)	Transferases	Glycerolipid metabolism	D0	2.02	0.001
EC 1.3.1.34	2,4-Dienoyl-CoA reductase [(2E)-enoyl-CoA-producing]	Oxidoreductases	lipid metabolism	D0	2.06	< 0.001
EC 2.7.1.121	Phosphoenolpyruvate-glycerone phosphotransferase	Transferases	Glycerolipid metabolism	D0	2.16	0.001
EC 3.1.4.46	Glycerophosphodiester phosphodiesterase	Hydrolases	Glycerophospholipid metabolism	D0	2.47	0.005
EC 2.1.1.37	DNA (cytosine-5-)-methyltransferase	Transferases	Cysteine and methionine metabolism	D0	2.73	< 0.001
EC 1.2.7.12	Formylmethanofuran dehydrogenase	Oxidoreductases	Methane metabolism	Week 20	2.34	< 0.001
EC 1.17.1.9	Formate dehydrogenase	Oxidoreductases	Methane metabolism	Week 20	2.32	< 0.001
EC 1.7.99.1	Hydroxylamine reductase	Oxidoreductases	Nitrogen metabolism	Week 20	2.41	< 0.001
EC 1.7.2.2	Nitrite reductase (cytochrome; ammonia-forming)	Oxidoreductases	Nitrogen metabolism	Week 20	2.39	0.014
EC 1.17.1.10	Formate dehydrogenase (NADP^+^)	Oxidoreductases	Methane metabolism	Week 20	2.14	< 0.001
EC 2.4.2.18	Anthranilate phosphoribosyltransferase	Transferases	Phenylalanine, tyrosine and tryptophan biosynthesis	Week 20	2.31	< 0.001
EC 3.5.1.2	Glutaminase	Hydrolases	Alanine, aspartate and glutamate metabolism,	Week 20	2.42	< 0.001
EC 4.2.1.33	3-Isopropylmalate dehydratase	Lyases	Valine, leucine and isoleucine biosynthesis	Week 20	2.25	< 0.001
EC 3.5.2.14	N-methylhydantoinase (ATP-hydrolysing)	Hydrolases	Arginine and proline metabolism	Week 20	2.18	< 0.001
EC 2.7.1.165	Glycerate 2-kinase	Transferases	Glycine, serine and threonine metabolism	Week 20	2.17	< 0.001
EC 5.4.99.5	Chorismate mutase	Isomerases	Phenylalanine, tyrosine and tryptophan biosynthesis	Week 20	2.06	< 0.001
EC 2.5.1.47	Cysteine synthase	Transferases	Cysteine and methionine metabolism	Week 20	2.05	0.020
EC 4.1.2.13	Fructose-bisphosphate aldolase	Lyases	Fructose and mannose metabolism	Week 20	2.04	0.024
EC 5.4.3.8	Glutamate-1-semialdehyde 2,1-aminomutase	Isomerases	Biosynthesis of secondary metabolites	Week 20	2.04	< 0.001
EC 6.4.1.1	Pyruvate carboxylase	Ligases	Pyruvate metabolism	Week 20	2.32	< 0.001
EC 2.4.1.10	Levansucrase	Transferases	Starch and sucrose metabolism	Week 20	2.22	< 0.001
EC 2.7.4.25	(d)CMP kinase	Transferases	Pyrimidine metabolism	Week 20	2.13	< 0.001
EC 6.3.4.2	CTP synthase (glutamine hydrolysing)	Ligases	Pyrimidine metabolism	Week 20	2.12	< 0.001
EC 3.5.4.2	Adenine deaminase	Hydrolases	Purine metabolism	Week 20	2.01	< 0.001
EC 4.1.99.17	Phosphomethylpyrimidine synthase	Lyases	Thiamine metabolism	Week 20	2.29	< 0.001
EC 3.2.1.93	Alpha,alpha-phosphotrehalase	Hydrolases	Starch and sucrose metabolism	Week 20	2.00	0.001

Furthermore, over the sampling periods we observed 25 substantially different genes encoding CAZymes (*P* < 0.05). Among these genes, GH20, GH3, GH28 and GT36 were abundant at baseline, while genes GT4, GH97, GT26 and CBM50 were prevalent at week 4, and three genes encoding GH95, GT19 and GH101 exhibited greater abundance at week 8. At week 12, four genes, namely GH31, GT30, GT28 and GT39, were significantly abundance. Lastly, seven genes encoding GHs (GH77, GH32, GH26, GH68, GH105, GH59 and GH4) and four genes encoding GTs (GT35, GT5, GT66 and GT25) were more abundant at week 20 (*P* < 0.05).

### Relationships between differentially abundant rumen microbiota and metabolic pathways

**Fig. 6:**
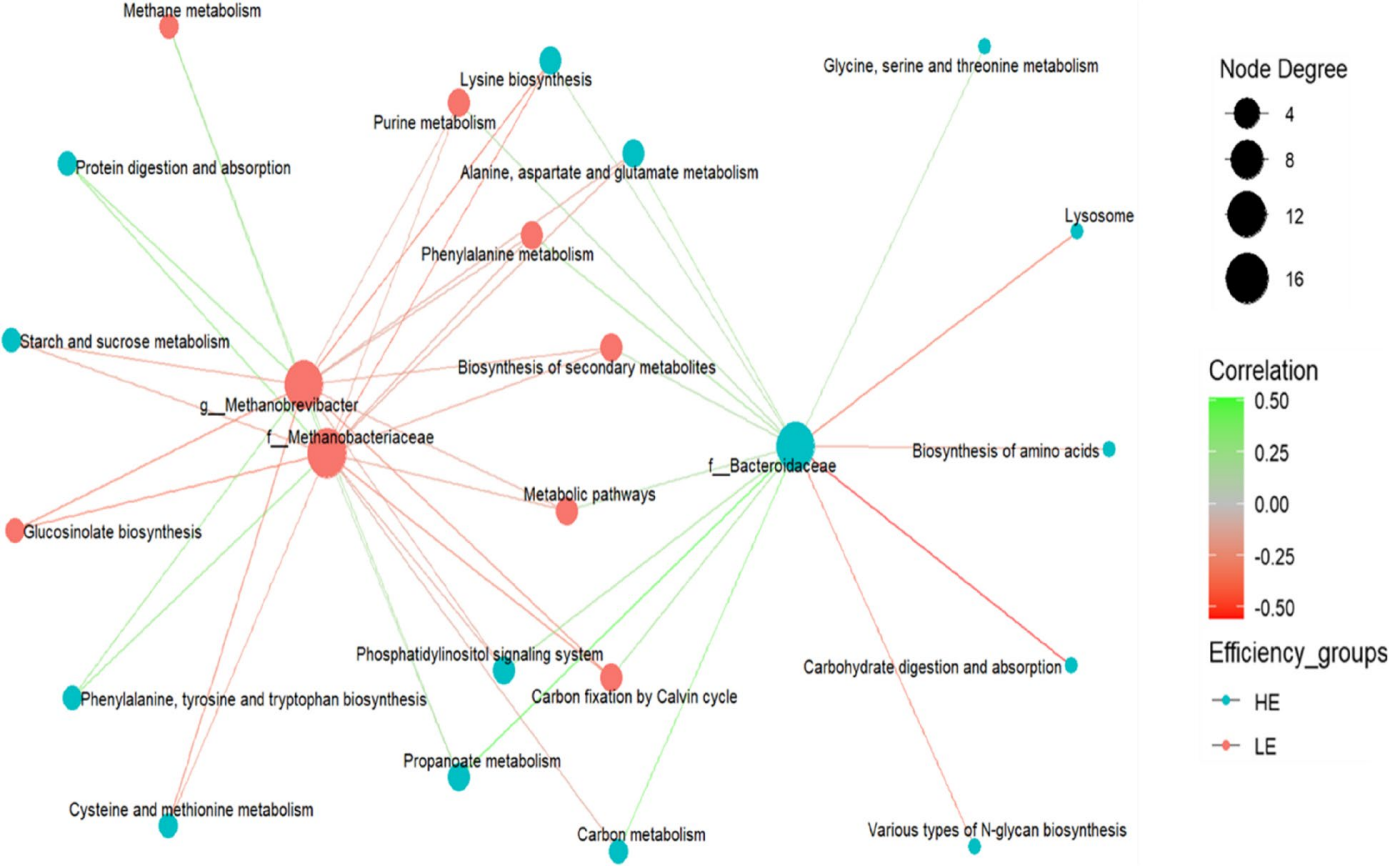
Correlation networks between differentially abundant microbial taxa and metabolic pathways. The size of the nodes indicates the number of connections (degree); large nodes have more connections (higher degree), while small nodes have fewer connections. Red nodes represent low-efficiency (LE) cows, and cyan nodes represent high-efficiency (HE) cows. Edges in red indicate a negative correlation between the taxa and metabolic pathway, while green edges indicate a positive correlation. Only moderately strong (coefficient *r* > 0.4 or < −0.4) and significant (*P *< 0.05) correlations were considered for further discussion

The functional interactions between significantly abundant rumen microbiota and metabolic pathways were assessed using Spearman’s rank correlation method. This analysis involved two microbial families (Bacteroidaceae and Methanobacteriaceae*)* as well as the genus *Methanobrevibacter* and 24 differentially abundant KEGG pathways. We found that the family Bacteroidaceae was positively correlated with eleven pathways (*P* < 0.05), such as ko00300 (lysine biosynthesis), ko00360 (phenylalanine metabolism), ko04070 (phosphatidylinositol signaling system), ko00230 (purine metabolism), ko00640 (propanoate metabolism), ko01200 (carbon metabolism), ko00260 (glycine, serine and threonine metabolism), ko00710 (carbon fixation by Calvin cycle), ko00250 (alanine, aspartate and glutamate metabolism), ko01110 (biosynthesis of secondary metabolites) and ko01100 (metabolic pathways), while ko04142 (lysosome), ko00513 (various types of N-glycan biosynthesis), ko04973 (carbohydrate digestion and absorption), ko01230 (biosynthesis of amino acids), and ko00500 (starch and sucrose metabolism) pathways were negatively correlated (Fig. 6). Among them, ko01200 (carbon metabolism), and ko00640 (propanoate metabolism) exhibited relatively strong positive correlations (with r values of 0.45 and 0.51, respectively). In contrast, ko00513 (various types of N-glycan biosynthesis), ko04142 (lysosome) and ko04973 (carbohydrate digestion and absorption) pathways had correlation coefficients of −0.43, −0.41, and −0.56, respectively (*P* < 0.01). The f__Methanobacteriaceae exhibited a significant positive association with ko04974 (protein digestion and absorption), ko00640 (propanoate metabolism), ko00400 (phenylalanine, tyrosine and tryptophan biosynthesis), and ko00680 (methane metabolism) pathways, whereas a negative association was detected with ko00300 (lysine biosynthesis), ko04070 (phosphatidylinositol signaling system), ko01200 (carbon metabolism), ko00360 (phenylalanine metabolism), ko00966 (glucosinolate biosynthesis), ko00270 (cysteine and methionine metabolism), ko00710 (carbon fixation by Calvin cycle), ko00500 (starch and sucrose metabolism), ko00250 (alanine, aspartate and glutamate metabolism), ko01110 (biosynthesis of secondary metabolites), and ko01100 (metabolic pathways). The *g__Methanobrevibacter* was inversely correlated with ko00300 (lysine biosynthesis), ko04070 (phosphatidylinositol signaling system), ko00360 (phenylalanine metabolism), ko00230 (purine metabolism), ko00966 (glucosinolate biosynthesis), ko00270 (cysteine and methionine metabolism), ko00710 (carbon fixation by Calvin cycle), ko00500 (starch and sucrose metabolism), ko00250 (alanine, aspartate and glutamate metabolism), and ko01100 (metabolic pathways), whilst a positive association was noticed with ko04974 (protein digestion and absorption), ko00640 (propanoate metabolism), ko00400 (phenylalanine, tyrosine and tryptophan biosynthesis) and ko00680 (methane metabolism) (Fig. 6). The methane metabolism pathway, which was upregulated in LE cows, was positively correlated with both f__Methanobacteriaceae and *g__Methanobrevibacter*.

## Discussions

The aim of this study was to evaluate whether it is possible to reduce the CP content of the dairy cow diet and achieve improved NUE and reduced environmental N (especially ammonia) release, which is a local pollutant and can also be indirectly converted to the potent greenhouse gas (GHG) nitrous oxide in soil and slurries. Our data suggest that CP content of dairy cow diets can be reduced from 17% to 15%, which enhances NUE and reduces environmental N extraction, whilst having no detrimental effects on the rumen microbiome.

### Dietary protein effects on feed intake and milk performance

Lavery et al. [22] have already discussed feed intake and milk production within this study, with key outcomes briefly reviewed here. In agreement with the findings of the current study, previous studies have found DMI to be unaffected when diet CP levels were reduced to around 15% in early lactation [14, 42, 43]. However, the impact of diet CP content on DMI is not always consistent with Broderick [44] finding a reduction in DMI when total diet CP was reduced from 18.4% to 15.1%, while Hymøller et al. [45] found that DMI was reduced when total diet CP was reduced from 16% to 14%. Reasons for differing responses between studies are not always clear, although feeding low dietary protein levels might restrict microbial growth affecting feed intake and digestion.

As with DMI, the impact of diet CP on milk yield has not always been consistent, with Law et al. [14] finding a reduction in milk yield at diet CP levels less than 15%, while Räisänen et al. [46] found no effect. Nevertheless, given the absence of an effect of treatment on DMI in the current study, it was perhaps unsurprising that milk yield was also unaffected. However, key to the design of the current study is the fact that all diets supplied the cows MP requirements, albeit with diets which differed in CP level. Thus, the current study suggests that cow performance can be maintained provided the diet meets the cows' MP requirements, even at a CP content of 15% (DM basis). In the majority of studies where both intake and milk production were unaffected by diet protein levels, milk fat and protein content was also unaffected, as in the current study.

### Dietary protein effects on nitrogen-use-efficiency and excretion patterns

Milk urea nitrogen is often considered to provide an indication of NUE in dairy cows. Increasing MUN content with increasing diet CP has been widely observed [47–49], with the magnitude of responses in these studies similar to that observed in the current study. MUN levels in cows between 10 and 12 mg/dL typically suggest optimal dietary N intake [50]; however, MUN levels over 12 mg/dL, as was the case in the HCP diet in the current study, indicate that the cow is squandering dietary N by excreting it as urinary urea N, as described by Kebreab et al. [51].

Nitrogen-use-efficiency in dairy cows is generally low, with Huhtanen and Hristov [52] reporting mean NUE values of 25% and 28% in North America and Northen Europe, respectively. While many strategies to improve NUE have been examined, including synchronising dietary N and energy supply in the rumen [53], reducing the protein content of the diet is perhaps most effective. In this study we noted that a 2% decrease in CP content of the diet to 15% significantly enhanced NUE in dairy cows. Other studies have also noted that decreasing diet CP content by 1% unit resulted in 2% [48], 3.3% [54] and 3.5% [49] improvements in NUE. In our study, NUE increased by approximately 9% following both a 1% and 2% reduction in dietary CP, which is a major enhancement.

The predicted N excretion in faeces was unaffected by dietary protein level in the current study and agrees with the findings of some other publications [55, 56], although not all previous studies [57]. In contrast, urinary N excretion was predicted to increase from 175 g/d with LCP to 231 g/d with HCP, with a reduction in urinary N excretion with decreasing diet CP levels having been observed in many studies involving a similar range in diet CP levels [27, 44, 58, 59]. With the LCP diet less N was excreted in urine than in faeces, in line with the findings of Dijkstra et al. [60]. The 27 g/d increase in predicted urine N excretion with every 1%-unit increase in total diet CP level is comparable to values of 27 and 24 g/d reported by Cressman et al. [61] and Groff and Wu [48], respectively, but lower than the value of 53 g/d reported by Broderick [44]. The increased excretion of N in urine (rather than faeces) with increasing diet CP levels reflects dietary N which is not captured by rumen microbes being converted to ammonia, which is subsequently converted to urea in the liver and excreted primarily in urine. This excess dietary N is also reflected in increasing MUN concentrations with increasing diet CP levels [22].

### Relationship between nitrogen-use-efficiency and residual feed intake

Within each treatment there was a clear negative relationship between NUE and RFI, cows with a high NUE having a low RFI (more efficient). Similar relationships were observed by Liu and VandeHaar [21] with cows offered diets differing in protein levels in both early and late lactation. In this latter study the correlation coefficient between RFI and NUE across diets was − 0.42 for early-lactation cows and − 0.24 for late-lactation cows, the values for early lactation cows being lower than observed in the current study (−0.57). We are unaware of any other study which has examined relationships between NUE and RFI in lactating dairy cows, therefore this data is novel. Nevertheless, Paul et al. [62] noted that selection for lower RFI could reduce manure N by 15%–17%. In contrast, Rius et al. [63] found no differences in N utilisation efficiency between growing heifers with high or low RFI, while Thornhill et al. [64] and Marett et al. [65] demonstrated that animals selected for lower RFI as calves or heifers did not have an improved NUE in the subsequent lactation. Given that the underlying mechanism between improved NUE and low RFI is not yet clear, this study was designed to establish whether this was linked to the rumen microbiome.

### Dietary protein effects on rumen fermentation parameters

The concentrations of VFAs, the principal fermentation products in rumen, are key indicators of rumen microbial activity. However, molar concentrations were unaffected by diet in this study. Similarly, molar concentrations were unaffected when diet CP increased from 135 to 194 g/kg DM [54] and from 130 to 175 g/kg DM [49]. Additionally, Belanche et al. [66] noted that production of the main rumen VFAs did not differ between dairy cows fed low-protein and high-protein diets. While Belanche et al. [66] found molar fractions of iso-butyrate and iso-valerate to be reduced with cows offered low protein diets, no such effect was observed in the current study. The results of the current study and published literature suggest that reducing diet CP within a certain range does not have a negative impact on rumen fermentation.

The greater ruminal-NH_3_-N concentration with the HCP diet in the present study aligns with the higher MUN concentrations observed and the greater predicted urinary N excretion. This reflects excess protein in the diet, as already discussed. Increasing NH_3_-N concentrations with increasing diet CP levels have been observed in many previous studies [66–68].

In common with the results of the current study, a few other studies have found rumen fluid pH to be unaffected by diet protein content [27, 54, 68–71]. Although the higher rumen ammonia N concentration with the HCP treatment might have been expected to increase rumen pH, this was not observed in the current study. Ruminal pH was unaffected by diet CP concentrations, while rumen pH with all diets was within the optimum pH range for microbial fermentation.

### Rumen microbial community composition, diversity, and differential abundance across diet CP concentrations

The phylum Bacteroidetes and Bacillota, along with the family Prevotellaceae and the genus *Prevotella,* were the dominant microbiota in this study, which is consistent with previous metagenome studies by Delgado et al. [72], Shabat et al. [73], and Xue et al. [39], who identified high abundances of those microbiota in rumen. Alpha diversity indices and PCoA results in the present study were unaffected in response to diet CP level. In contrast, Zhang et al. [74] using 16S rRNA sequencing observed that the high-protein diet (13.87% CP) reduced alpha diversity indices of bacterial communities, while archaea and fungi communities were more stable and resilient to treatment compared with the bacterial communities [75].

Differential abundance of microbiota across diets remained unchanged, agreeing with Zhou et al. [76], who reported similar abundances of the phylum (Firmicutes and Bacteroidetes*)* and the genus (*Prevotella_1, Christensenellaceae_R-7_group, Fibrobacter, Ruminococcus_1, Butyrivibrio_2)* among beef cattle on a low CP diet (111 g/kg DM) vs. on a high CP diet (136 g/kg DM). However, in dairy cows, studies comparing the effects of various CP levels on microbial composition and functions using both shotgun and 16S rRNA sequencing techniques are less documented. Studies in other animals like beef cattle [77], lambs [78] and goats [79] reported mixed results. For example, He et al. [77] observed more *Butyrivibrio fibrisolvens* in high CP diet (133 g/kg DM) than in low CP diet (122 g/kg DM), but Lv et al*.* [78] detected higher Bacteroidetes abundance in lambs given high CP diets on lower dietary energy conditions, while Zhu et al. [79] found that the *Prevotella, Campylobacter, Synergistetes* and *TG5* were less abundant in goats on low CP diet (12.0%) than high CP diet (14.8%); no such variation was found between the diets in this study. Wang et al. [80] recently found that protein restriction reduced the abundances of the genera *Bacteroides, Prevotella,* and *Bifidobacterium*. Differences in species studied (cows, beef, goats, and lambs), variations in sequencing techniques and bioinformatics pipelines, and diet composition (energy levels) could be attributed to these inconsistent results. Indeed, despite the inconsistency of microbiome results across studies, the present results suggest that diet CP can be reduced to a level more sustainable in dairy cow diets without compromising microbial ecosystems, as well as the productivity of the host animal.

### Rumen microbial community composition, diversity, and differential abundance between HE and LE cows

We found that HE cows consumed on average 1.27 kg less feed than LE cows with similar milk production, which agrees with previous works [21, 81, 82]. The results of alpha and beta diversity together indicated that the overall composition of microbial community at the phylum, family, and genus levels was relatively unaffected between HE and LE cows. These findings agree with those of Li and Guan [83], who reported that animals with high and low-RFI values have comparable microbial richness, diversity and evenness. In contrast, other studies [73, 81] reported a greater richness in high residual feed intake (HRFI) cows.

While microbial differential abundance was similar between the two efficiency groups, more detailed LDA analysis showed that f__Bacteroidaceae was more abundant in HE cows, whereas f**__**Methanobacteriaceae and *g__Methanobrevibacter* were more abundant in LE cows under specific dietary and temporal circumstances. Similarly, relatively high abundances of *Methanosphaera* [81], *Methanobrevibacter ruminantium* [73] and *Methanobrevibacter* [72] were noted in HRFI cows. Other studies also reported a positive association between *Methanobrevibacter SGMT clade* and CH_4_ emission [84–87]. These increased abundances of *Methanobrevibacter* might suggest that LE cows harbor a microbial profile that contributes to higher methane emissions, potentially affecting feed efficiency and energy utilisation, supporting the notion that enteric CH_4_ production results in energy loss of approximately 2%–12% during ruminal digestion, which contributes approximately 6% of global anthropogenic GHG emissions [88].

### Rumen microbial community composition, diversity, and differential abundance over the sampling periods

Our results provide a comprehensive overview of temporal dynamics of the rumen microbial community composition at various taxonomic levels. The current results illustrate substantial changes in microbial richness, evenness and diversity over time, indicating variations in the relative abundance of specific taxa over time. Consistently, earlier studies have highlighted that the rumen microbial community composition might be significantly influenced by variations in rumen sampling intervals ranging from hours to years [89]. In contrast, Li et al. [90] found no significant variations in bacterial composition when comparing samples collected three hours before feeding with those collected three- and nine-hours post-feeding. Compared with other periods, increased exposure to diets with varying CP levels resulted in a greater abundance of the phylum Euryarchaeota and Fibrobacterota and an unknown phylum from the order bacteria at week 20, highlighting the importance of long-term studies for better understanding microbial dynamics over time. In line with this, Bainbridge et al. [18] examined shifts in rumen bacterial communities across various days in milk (DIM) [3, 93, 183 and 273 DIM] and found that Bacteroidetes, Firmicutes and Proteobacteria were the most divergent phylum across DIM.

We also detected increased abundance of the family Lachnospiraceae, Clostridiaceae and Erysipelotrichaceae at baseline, where the Lachnospiraceae family contains members of bacteria associated with plant degradation and butyrate production in the gut [91]. Additionally, members of different families, including Bacteroidaceae, Fibrobacteraceae and Veillonellaceae varied in abundance over time. Golder et al. [92] mentioned that members of the family Veillonellaceae are known producers of propionate as their major fermentation product. An increased abundance of *g__Blautia* and *g__Butyrivibrio* at baseline reflect the baseline microbial composition and fermentation activities, consistent with Bainbridge et al. [18], who observed greater abundance of *Butyrivibrio* at early lactation (3 DIM) compared to 93, 183 and 273 DIM. Indeed, significant abundance of many genera, including *g__Methanobrevibacter* and *g__Fibrobacter* at week 20 suggested long-term adaptation to changes in the rumen environment and diet, with these genera playing an important role in methanogenesis and fibre degradation under specific dietary conditions. Overall, our results demonstrated that rumen microbiota composition and abundance is dynamic and sensitive to changes over time.

### The functional profiles of rumen microbial community across diet CP concentrations

The overall abundance of second-level functional categories and third-level KEGG pathways did not differ among diet CP concentrations. In contrast, Li et al. [93] found that some functional categories, including “carbohydrate transport and metabolism", “translation, ribosomal structure and biogenesis", “cell motility", and “cytoskeleton” were highly expressed at the RNA level compared with their DNA abundances, consistent with previous metatranscriptome studies [83]. This lack of differences in pathway and module abundance across diets in this study also highlights the importance of lowering CP levels (from 17% to 15% in our case) without altering metabolic functions. In contrast, Mao et al. [94] found enrichment of nucleotide metabolism and translation and a lower abundance of amino acid, carbohydrate, and energy metabolism in goats with a high concentrate:forage ratio (80:20, H) than in those with a low concentrate:forage ratio (25:75, L). Recently, Amin et al. [95] reported a greater prevalence of genes involved in N metabolism and lysine biosynthesis pathways in high-milk protein groups than in low-milk protein groups. Our results collectively denote static functional networks within the rumen microbial communities regardless of diet CP concentration.

Rumen microbes secrete several microbial enzymes, including exoglucanases, endoglucanases, glucosidases, and hemicellulases, to deal with complex plant polysaccharides, which are grouped into CBMs, GHs, GTs, PLs, and CEs [96]. GHs represent one of the largest enzyme families that hydrolyse reactions related to the metabolism of various polysaccharides, including starch, cellulose, xylan, and chitin [97], which was consistent with our study. The present results were also consistent with those of Lairson et al. [98], who reported that GTs were the second most abundant CAZy family identified that catalyse the activated oligosaccharide or glycosidic bonds to a variety of receptors, such as proteins, nucleic acids, lipids, and small molecules. Moreover, PLs, which cleave glycosidic bonds in acidic polysaccharides, were the least abundant CAZyme class in the cow rumen, and this result was consistent with that of Jose et al. [99]. In this study, CAZyme abundance was similar between diet CP levels, consistent with Wang et al. [100], who detected comparable abundances of certain genes encoding cellulases, hemi-cellulases, oligosaccharide-degrading enzymes, and debranching enzymes regardless of diet and sampling period. Similarly, Sato et al. [101] found similar abundance of cellulose-associated enzymes (GH5 or GH9) in Japanese black cattle and F1 steers. The present results illustrate that the concentration of CP in the diet can be reduced without disrupting enzymes involved in various metabolic functions within the host.

### Comparative functional profiles of the rumen microbial community between HE and LE cows

The functional profiles of the rumen microbiomes in cows with differing RFI were also previously assessed using metagenomics [73, 81, 93] and metatranscriptomics [83]. While microbial functions among HE and LE cows did not change in this study, multiple pathways, modules and enzymes differed under the same dietary and temporal conditions. HE cows exhibited the prevalence of various metabolic pathways and modules related to amino acid, energy, carbohydrate metabolism, and glycan biosynthesis along with enzymes involved in these pathways (EC 4.2.1.33, EC 4.2.1.35, EC 2.3.1.31, EC 3.3.1.1, EC 3.4.21.1 and EC 3.4.17.19), indicating better amino acid utilisation, which in turn meant more effective protein synthesis and improved energy utilisation. This might partly explain higher NUE in HE cows, supporting the rationale that metabolisable protein utilisation for milk protein and mammary amino acid (AA) synthesis are more efficient in cows with low-RFI than in the high-RFI cows [102]. Our observations were in line with those of Lima et al. [103], who identified some genes related to carbohydrate metabolism as being predictive of feed efficiency in beef cattle.

Moreover, upregulation of propanoate metabolism along with two genes encoding EC 1.2.1.18 and EC 1.2.1.27 in the rumens of HE cows potentially indicates more hydrogen utilisation and formation of substrates with greater usable energy, which agrees with Xie et al*.* [81], who detected more genes associated with propionate production in LRFI cows. Likewise, an increased proportion of propionate and propionate-to-acetate ratio was observed in high-and-medium-NUE cows than low-NUE cows [20] as well as feed efficient cows [73] than inefficient cows, this increased ratio was associated with reduced CH_4_ production and enhanced energy retention by cattle [104].

In contrast, metabolic pathways and modules related to CH_4_ metabolism, fatty acid metabolism, purine metabolism, cofactor and vitamin biosynthesis, and secondary metabolite biosynthesis with different enzymes associated with these functions were more abundant in LE cows. In agreement with these findings, increased abundances of CH_4_ metabolism and KEGG Orthology (KO) genes related to methanogenesis [81], and methanogenesis pathways [73] were detected in HRFI cows. Consistently, enrichment of “glycolysis”, “pentose phosphate pathway” and “fructose and mannose metabolism” [81], as well as amino acid metabolism pathways [83], has been reported in HRFI animals. A metagenomic study also showed enrichment of different pathways in HRFI animals [83, 105], which is consistent with the findings of Xie et al. [81], who reported less diverse metabolic pathways in efficient dairy cows. These inconsistencies across studies suggest that rumen microbial functions might be highly dynamic and variable, regardless of the efficiency status of the animal. This study highlights the importance of understanding the metabolic basis of feed efficiency and NUE, as it can lead to nutritional intervention to improve livestock production efficiency.

Our results revealed that the most abundant CAZymes GH13, GH3, and GH31 did not vary between HE and LE cows, while significant variations were observed for less abundant CAZyme. Specifically, GH77 and GT66 were abundant in HE cows, which was consistent with the findings of Tapio et al. [105], who identified relatively high levels of GT66, GT5 and GT2 in LRFI cows, whereas GH3, GH13, GH43, and GT51 were enriched in HRFI cows. In contrast to those in HE cows, GT4 and GH59 were highly abundant in LE cows, which is in agreement with the findings of Xie et al. [81], who observed a high number of genes encoding CAZymes in cows with HRFI. Indeed, the microbial differences found at the taxonomic and functional levels between the two groups of cows could explain the mechanisms underlying variations in their efficiency.

### Functional profiles of the rumen microbial community over the sampling periods

The current findings revealed that microbial functions varied over the sampling periods. For example, pathways and modules related to carbohydrates, nucleotide metabolism, glycan biosynthesis and metabolism, replication, and repair as well as enzymes involved in those processes were abundant at baseline. We also found higher abundances of ko00250, ko00460, ko00010, ko00051, ko00910, ko00190 and enzymes which catalyze different chemical reactions in those processes at week 4, supporting Kamra [106], who reported that microbes in the rumen secrete a group of fibrolytic enzymes, such as endoglucanases (EC 3.2.1.4), exoglucanases (EC 3.2.1.91), beta-glucosidases (EC 3.2.1.21), xylanases (EC 3.2.1.8), and lignin peroxidase (EC 1.11.1.14), which convert lignocellulose in the diet into VFAs. These results showed the dynamic nature of microbial functions over time and their ability to adapt to various factors, like dietary adjustments and changes in the rumen environment or host physiology over time.

The abundance of CAZymes was dynamic over time. For instance, GH20, GH3, GH28 and GT36 were abundant at baseline, whereas GT4, GH97, GT26 and CBM50 were more abundant at week 4. We also found that genes encoding cellulases predominantly belong to GH26, GH28, and GH95 families, while those encoding oligo-saccharide degrading enzymes belong to GH3, GH4, GH31, GH32, GH20, GH97, and GH68 families, which were also reported in earlier studies [100, 107, 108]. The genes encoding debranching enzymes were mainly represented in the GH77 family, as reported in a previous study by Wang et al. [100]. It is unclear why these discrepancies exist, but a possible explanation is that changes in diet composition and nutrient availability over time could contribute to these variations.

### Relationships between differentially abundant rumen microbiota and metabolic pathways

Our findings revealed that the family Bacteroidaceae is positively correlated with metabolic pathways, such as carbon, propanoate and phenylalanine metabolism. These positive correlations are consistent with the functional role of many members of the Bacteroidaceae family*,* which are the major fibre-degrading bacteria in the rumen. This is supported by Jami et al. [16], who stated that bacteria from the phylum Bacteroidetes*,* which includes Bacteroidaceae, are key contributors to carbohydrate degradation and propionate production in the rumen. Stevenson and Weimer [109] reported that Bacteroidetes are known for efficient starch, pectin and xylan digestion, while Firmicutes*,* Proteobacteria and Actinobacteria are known for cellulose and hemicellulose digestion. This positive correlation highlights the importance of these ruminal bacteria in enhancing energy utilisation efficiency of ruminants through efficient fibre breakdown and increased propionate production. Although the negative correlation between f__Bacteroidaceae and metabolic pathways (N-glycan biosynthesis and carbohydrate digestion and absorption) was unusual, this correlation could be due to a highly competitive metabolic environment favouring f__Bacteroidaceae fermentation over their participation in these pathways. Xue and coworkers [110] reported that members of family Bacteroidaceae—including *Succinivibrio* and *Prevotella—*were positively correlated with acetate, propionate and valerate in low-milk protein cows, whereas *Sharpea* was positively correlated with propionate and valerate concentrations in high-milk protein cows.

As expected, f__Methanobacteriaceae and *g__Methanobrevibacter* are positively associated with the CH_4_ metabolism pathway, as these microbes are known to be the primary methanogens in the rumen. The members of the genus *Methanobrevibacter* are well known producers of enteric CH_4_ through the reduction of hydrogen and carbon dioxide produced by other microbes [37]. While propionate production consumes H_2_, thereby reducing its availability for methanogens, the logical explanation for the intriguing positive correlation between propionate metabolism and methanogens in our study is not clear but could be speculated due to the possible syntrophic relationships between propionate-oxidising bacteria and hydrogen/formate- and acetate-utilising methanogens [111], where propionate could be breakdown to release hydrogen and other substrates, which could be utilised by methanogens. Thus, the present findings provide insights into our understanding of rumen microbial ecology, and functional networks and their impacts on ruminant metabolism and environmental emissions.

## Conclusions

Our data from these experiments show that the CP content of dairy cow diets can be reduced from 17% to 15% to enhance NUE and reduce environmental N extraction, whilst having no detrimental effects on the rumen microbiome. In addition, NUE was negatively correlated with RFI, implying that low-RFI cows showed higher NUE. Overall, microbial composition, diversity, and functional profiles provide strong evidence that reducing diet CP levels in dairy cows does not result in any obvious trade-offs. This study also revealed that HE and LE dairy cows differed in their rumen microbiome, with propanoate, cysteine and methionine metabolism being greater in HE cows than in LE cows. Overall, these results provide valuable insights into the potential benefits of optimising dietary CP concentrations in dairy cow diets, particularly in reducing N excretion while maintaining production efficiency, whilst also defining the underlying rumen microbiome associated with HE and LE cows.

## Supplementary Information

Additional file 1: Production and fermentation data.

Additional file 2: Taxonomic and functional data.

Additional file 3: Table S1 The ingredient composition of concentrate offered as part of diets designed to supply 15%, 16%, and 17% CP of total diet DM. Table S2 Chemical composition of grass silage and concentrates in the diets of dairy cows offered diets differing in CP concentrations. Table S3A PERMANOVA (permutational multivariate analysis of variance) of phylum, family and genus between diet CP levels. Table S3B PERMANOVA (permutational multivariate analysis of variance) of phylum, family and genus between efficiency groups. Table S3C PERMANOVA (permutational multivariate analysis of variance) of phylum, family and genus between sampling periods. Fig. S1 Stacked bar chart showing theoretical and NGS measured microbial composition in positive control (ZymoBIOMICS microbial community standard). Fig. S2 PCoA plot of the rumen microbial compositional profiles across diet (A) and efficiency groups (B) at the phylum level (*n* = 177). Fig. S3 PCoA plot of the rumen microbial compositional profiles across treatment (A) and efficiency groups (B) at the family level (*n* = 177).

## Data Availability

The raw read data is available on the European Nucleotide Archive (ENA) under project number ERP149388.

## Abbreviations

AA: Amino acid
AA: Auxiliary activity
AFBI: Agri-food and biosciences institute
BH: Benjamini‒Hochberg
BW: Body weight
CH_4_: Methane
CP: Crude protein
COGs: Clusters of orthologous groups
CAZymes: Carbohydrate active enzymes
CBMs: Carbohydrate-binding modules
CEs: Carbohydrate esterases
DM: Dry matter
DMI: Dry matter intake
DIM: Days in milk
EC: Enzyme Commission
ECM: Energy corrected milk
GHG: Greenhouse gas
RFI: Residual feed intake
FiM: Feed into milk
GCTU: Genomics core technology unit
GH: Glycoside hydrolase
GT: Glycoside transferase
HCP: High crude protein
HE: High efficiency cows
HRFI: High residual feed intake
HSD: Honest significant difference
KEGG: Kyoto Encyclopedia of Genes and Genomes
KO: KEGG orthology
LCP: Low crude protein
LDA: Linear discriminant analysis
LE: Low efficiency cows
LefSe: Linear discriminant effect size
LRFI: Low residual feed intake
MCP: Medium crude protein
MP: Metabolisable protein
MUN: Milk urea nitrogen
NUE: Nitrogen use efficiency
N: Nitrogen
NH_3_-N: Ammonia nitrogen
NIRS: Near infrared reflectance spectroscopy
PERMANOVA: Permutational multivariate analysis of variance
PCoA: Principal coordinate analysis
PLs: Polysaccharide lyases
REML: Restricted maximum likelihood
RMA: Reduced major axis
TMM: Trimmed mean of M-values
VFA: Volatile fatty acid
