# Supplementary material for: Reducing crude protein content in the diet of lactating dairy cows improved nitrogen-use-efficiency and reduced N excretion in urine, whilst having no obvious effects on the rumen microbiome
Source: J Anim Sci Biotechnol. 2025 Aug 9;16:113. doi: 10.1186/s40104-025-01240-7 (PMC12335128; doi:10.1186/s40104-025-01240-7)
Supplement: Supplementary file 3 — Additional file 3: Table S1 The ingredient composition of concentrate offered as part of diets designed to supply 15%, 16%, and 17% CP of total diet DM. Table S2 Chemical composition of grass silage and concentrates in the diets of dairy cows offered diets differing in CP concentrations. Table S3A PERMANOVA (permutational multivariate analysis of variance) of phylum, family and genus between diet CP levels. Table S3B PERMANOVA (permutational multivariate analysis of variance) of phylum, family and genus between efficiency groups. Table S3C PERMANOVA (permutational multivariate analysis of variance) of phylum, family and genus between sampling periods. Fig. S1 Stacked bar chart showing theoretical and NGS measured microbial composition in positive control (ZymoBIOMICS microbial community standard). Fig. S2 PCoA plot of the rumen microbial compositional profiles across diet (A) and efficiency groups (B) at the phylum level (n = 177). Fig. S3 PCoA plot of the rumen microbial compositional profiles across treatment (A) and efficiency groups (B) at the family level (n = 177). [file 40104_2025_1240_MOESM3_ESM.docx]

**Table S1** The ingredient composition of concentrate offered as part of diets designed to supply 15%, 16%, and 17% CP of total diet DM

|  | **Target total diet crude protein levels** | | |
| --- | --- | --- | --- |
| **Ingredient, %** | **15%** | **16%** | **17%** |
| Maize | 77.9 | 68.3 | 58.7 |
| Sugar-beet pulp | 32.0 | 32.0 | 32.0 |
| Wheat | 27.5 | 23.0 | 18.6 |
| Barley | 16.8 | 17.1 | 17.5 |
| Soyahulls | 27.4 | 22.7 | 18.0 |
| Soya-bean meal | 23.5 | 23.3 | 22.9 |
| Protected protein (Sopralin) | 6.3 | 7.0 | 12.5 |
| Rape-seed meal | 20.2 | 26.2 | 32.3 |
| Distillers dark grains | 4.4 | 11.9 | 19.4 |
| Mineral/Vitamin pre-mix | 7.0 | 7.2 | 7.4 |
| Protected fat (Megalac) | 5.6 | 5.6 | 5.6 |
| Molasses | 5.0 | 5.0 | 5.0 |
| Dicalcium phosphate | 1.0 | 0.6 | 0.3 |

Values presented represent the mean ingredient composition (weighted for the mean number of days each concentrate was offered for) of each concentrate offered during the experiment

**Table S2** Chemical composition of grass silage and concentrates in the diets of dairy cows offered diets differing in CP concentrations

|  |  | **Concentrates offered within each treatment** | | |  |
| --- | --- | --- | --- | --- | --- |
|  | **Grass silage** | **15%** | **16%** | **17%** | **Parlour concentrate^1^** |
| Oven DM, g/kg | 356 (57.3) | 885 (6.1) | 886 (6.8) | 888 (6.3) | 890 (6.2) |
| VCODM, g/kg | 367 (56.6) | - | - | - | - |
| Ash, g/kg DM | 81 (2.8) | 79 (6.7) | 79 (8.4) | 76 (7.7) | 76 (1.3) |
| ADF, g/kg DM | 259 (19.4) | 122 21.6) | 133 (23.9) | 138 26.3) | 126 16.1) |
| NDF, g/kg DM | 471 (23.2) | 223 29.8) | 245 (31.1) | 263 38.6) | 264 22.1) |
| Starch, g/kg DM |  | 337 37.8) | 281 (35.2) | 234 29.5) | 290 24.6) |
| GE, MJ/kg DM | 18.6 (0.67) | 18.0 (0.21) | 18.2 (0.18) | 18.5 (0.21) | 18.4 (0.09) |
| ME, MJ/kg DM^2^ | 12.0 (0.32) | 12.3 (0) | 12.3 (0.1) | 12.3 (0.1) | 12.3 (0) |
| Nitrogen, g/kg DM | 21.4 (1.96) | 26.8 (1.77) | 30.2 (2956) | 32.7 (2.40) | 28.5 (2.44) |
| pH | 4.0 (0.26) | - | - | - | - |
| Lactic acid, g/kg DM | 91 (33.8) | - | - | - | - |
| Acetic acid, g/kg DM | 14 (5.8) | - | - | - | - |
| Ethanol, g/kg DM | 10 (4.4) | - | - | - | - |
| Ammonia-N, g/kg N | 62 (11.8) | - | - | - | - |

Values in the bracket represent standard deviation

^1^Commercial concentrate ‘0.25’ kg offered at each milking for all cows

Abbreviation: VCODM=Volatile Corrected Oven Dry Matter, ADF=Acid Detergent Fibre, NDF=Neutral Detergent Fibre, GE = gross energy, ME = metabolizable energy content. ^2^Metabolizable energy (ME) content of grass silage was predicted using NIRS, and ME content of concentrates was calculated using DietCheck ration formulation software (DietCheck Advanced v10.1, DietCheck Ltd, Lancaster, UK)

**Table S3A** PERMANOVA (permutational multivariate analysis of variance) of phylum, family and genus between diet CP levels

| **Taxonomy** | **SumsOfSqs** | **MeanSqs** | **F. Model** | **R2** | ***P*. adjust** |
| --- | --- | --- | --- | --- | --- |
| Phylum | 0.0654 | 0.0327 | 1.2742 | 0.01418 | 0.243 |
| Family | 0.0950 | 0.0475 | 1.1709 | 0.01308 | 0.274 |
| Genus | 0.1249 | 0.0625 | 1.1518 | 0.01270 | 0.286 |

**Table S3B** PERMANOVA (permutational multivariate analysis of variance) of phylum, family and genus between efficiency groups

| **Taxonomy** | **SumsOfSqs** | **MeanSqs** | **F. Model** | **R2** | ***P*. adjust** |
| --- | --- | --- | --- | --- | --- |
| Phylum | 0.0558 | 0.0558 | 2.1769 | 0.01212 | 0.410 |
| Family | 0.0809 | 0.0809 | 1.9953 | 0.01114 | 0.450 |
| Genus | 0.1558 | 0.1558 | 2.8751 | 0.01585 | 0.095 |

**Table S3C** PERMANOVA (permutational multivariate analysis of variance) of phylum, family and genus between sampling periods

| **Taxonomy** | **SumsOfSqs** | **MeanSqs** | **F. Model** | **R2** | ***P*. adjust** |
| --- | --- | --- | --- | --- | --- |
| Phylum | 0.3770 | 0.0943 | 3.6752 | 0.08182 | 0.006 |
| Family | 0.6287 | 0.1572 | 3.8758 | 0.08656 | 0.001 |
| Genus | 0.8630 | 0.2158 | 3.9807 | 0.08779 | 0.001 |


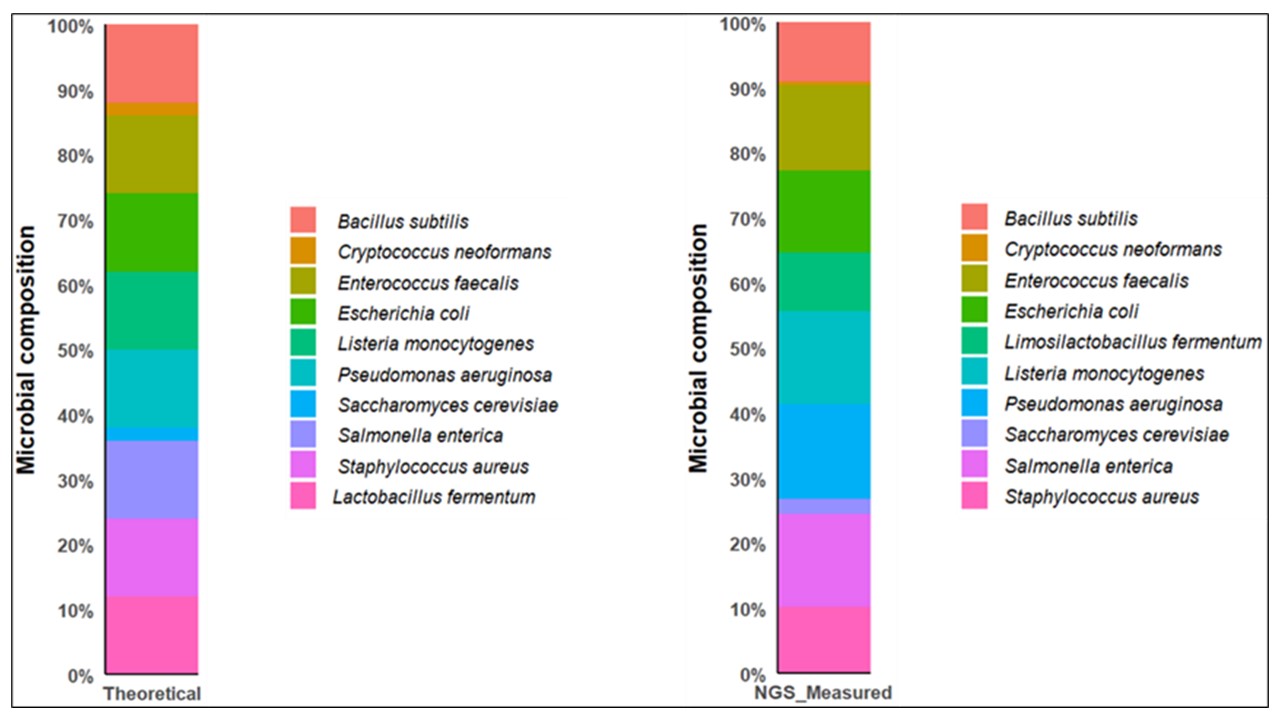


**Fig. S1** Stacked bar chart showing theoretical and NGS measured microbial composition in positive control (ZymoBIOMICS microbial community standard)


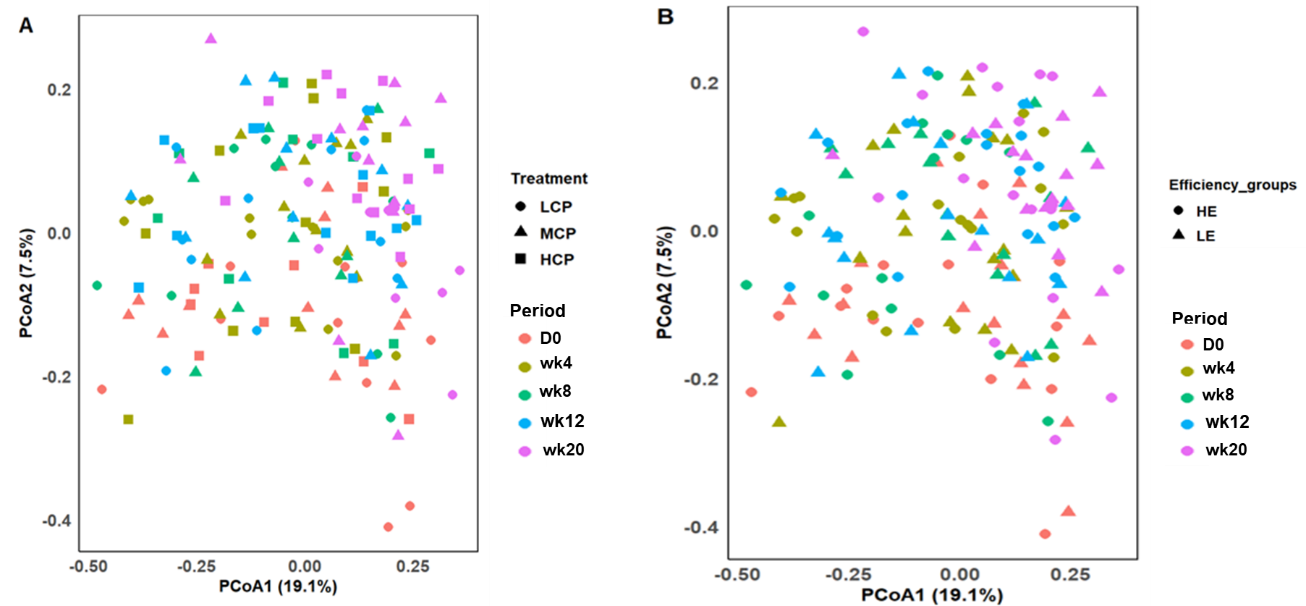


**Fig. S2** PCoA plot of the rumen microbial compositional profiles across diet (**A**) and efficiency groups (**B**) at the phylum level (*n* = 177). Periods: D0 (baseline); wk4 (week 4); wk8 (week 8); wk12 (week 12) and wk20 (week 20), Treatment: LCP: 15% CP, MCP: 16% CP, HCP: 17% CP, Efficiency groups (HE: high-efficiency cows, LE: low-efficiency cows)

**
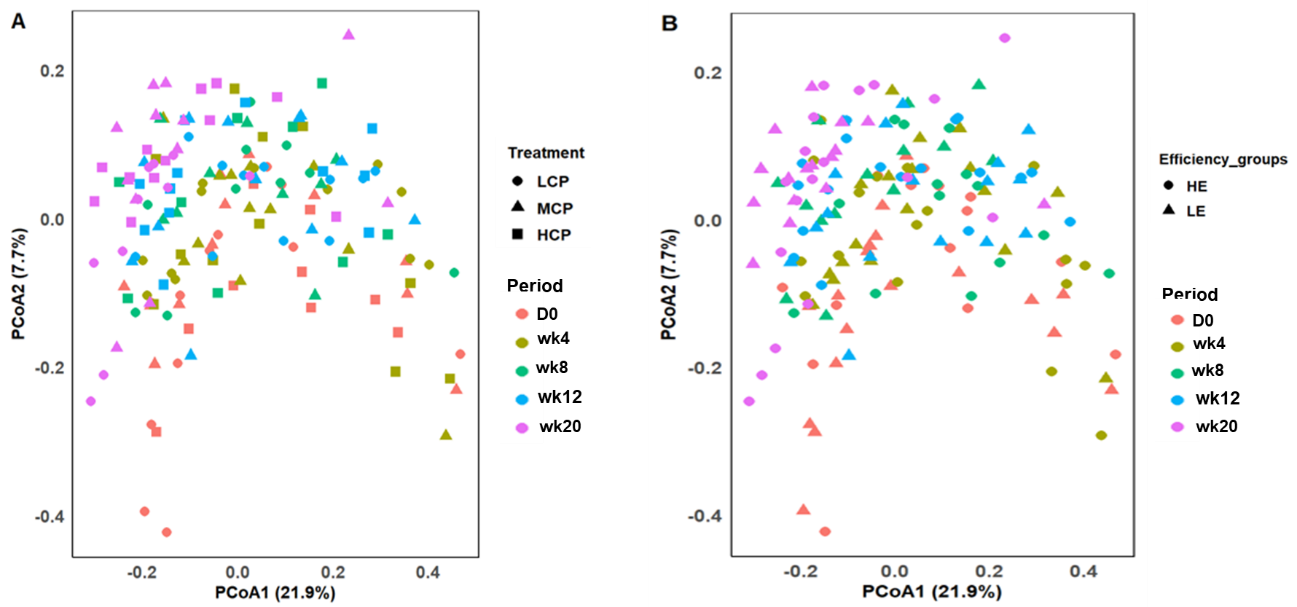
**

**Fig. S3** PCoA plot of the rumen microbial compositional profiles across treatment (**A**) and efficiency groups (**B**) at the family level (*n* = 177). Periods: D0 (baseline); wk4 (week 4); wk8 (week 8); wk12 (week 12) and wk20 (week 20), Treatment: LCP: 15% CP, MCP: 16% CP, HCP: 17% CP, Efficiency groups (HE: high-efficiency cows, LE: low-efficiency cows)
